# Developmental and Seasonal Changes in Lipid Droplets and Fatty Acid Composition in the Ovary and Liver of Female Spotted Scat (*Scatophagus argus*)

**DOI:** 10.3390/ani16050748

**Published:** 2026-02-27

**Authors:** Honglin Chen, Guangli Li, Yucong Hong, Chunhua Zhu, Huapu Chen, Siping Deng, Dongneng Jiang, Mouyan Jiang, Changxu Tian, Tuo Wang

**Affiliations:** 1 Fisheries College, Guangdong Ocean University, Zhanjiang 524088, China; 15642277985@stu.gdou.edu.cn (H.C.);; 2 Guangdong Research Center on Reproductive Control and Breeding Technology of Indigenous Valuable Fish Species, Guangdong Ocean University, Zhanjiang 524088, China; 3 Guangdong Provincial Engineering Laboratory for Mariculture Organism Breeding, Guangdong Ocean University, Zhanjiang 524088, China; 4 Guangdong Provincial Key Lab of Pathogenic Biology and Epidemiology for Aquatic Economic Animals, Guangdong Ocean University, Zhanjiang 524088, China; 5 Guangdong Provincial Key Laboratory of Aquatic Larvae Feed, Jieyang 515500, China

**Keywords:** fatty acids, lipid droplets, liver, ovary, broodstock, spotted scat

## Abstract

The spotted scat (*Scatophagus argus*) is a fish species widely distributed in Indo-Pacific waters. In China, females are preferred for aquaculture due to their faster growth rates. Reproduction in teleost fish requires substantial energy, primarily in the form of lipids and fatty acids, which are closely linked to reproductive success and ovarian development. In this study, female spotted scat broodstock were sampled bi-monthly from May 2019 to March 2020 to examine annual ovarian development, lipid droplet accumulation, and fatty acid composition in the liver and ovaries. Body weight, total length, standard length, and condition factor (CF) increased gradually over the sampling period. In July 2019, the gonadosomatic index (GSI) increased significantly, all ovaries reached stage IV, and lipid droplet accumulation was most pronounced. Ovarian saturated fatty acids (SFA), monounsaturated fatty acids (MUFA), n-3 polyunsaturated fatty acids (n-3 PUFA), and n-6 PUFA reached their highest levels in July, whereas the ovarian n-3 PUFA/n-6 PUFA ratio was highest in May. The hepatosomatic index (HSI) was significantly higher from November to March than from May to September. The viscerosomatic index (VSI) was significantly elevated from November to March and in July. Liver lipid droplet accumulation was lowest from May to July. Absolute contents of liver SFA, MUFA, and n-6 PUFA were significantly higher in January and March than in May and July, while liver n-3 PUFA content and the n-3 PUFA/n-6 PUFA ratio peaked in May. These findings indicate that the peak breeding season of spotted scat occurs around July in southern China. From winter to spring, lipids accumulate in the abdominal cavity and liver, likely serving as energy reserves for subsequent ovarian development. The liver appears to play a key role in the uptake and transfer of n-3 long-chain polyunsaturated fatty acids (n-3 LC-PUFA) to the ovaries prior. This study provides useful insights into the reproductive physiology of spotted scat and supports improved broodstock management in aquaculture.

## Simple Summary

Reproductive success in fish depends strongly on the availability and distribution of energy reserves, particularly lipids and fatty acids. In aquaculture, understanding how these nutrients change during ovarian development is essential for improving broodstock management. In this study, female spotted scat were sampled every two months over the course of one year to examine changes in ovarian development, lipid droplet accumulation, and fatty acid composition in the liver and ovaries. The results showed that ovarian maturation occurred mainly in summer, accompanied by a marked increase in lipid droplets and fatty acids in the ovary. In contrast, lipid droplets and fatty acids accumulated in the liver during winter and spring, suggesting energy storage before reproduction. These findings suggest that the liver plays an important role in storing and supplying fatty acids for ovarian development. This study provides useful information for improving nutritional strategies and reproductive management of female spotted scat in aquaculture.

## 1. Introduction

Fish reproduction is generally seasonal, and understanding annual reproductive cycles is fundamental for the artificial reproduction of cultured fish. Ovarian development in teleosts can be divided into early differentiation, maturation, and ovulation. Early ovarian development in bony fish is regulated by genetic factors as well as environmental factors such as temperature and photoperiod [1,2]. Physiological responses and nutritional requirements during the reproductive period differ substantially between broodstock and juvenile fish [3]. Oocyte maturation is accompanied by yolk accumulation, which consists mainly of proteins and lipids [4]. In teleost fish, the coalescence (fusion) of oil droplets during oocyte maturation is a well-established morphological indicator of final maturation stage and egg quality in various species [5]. The liver plays a crucial role in vitellogenin synthesis, and ovarian development involves the transfer of nutrients from somatic tissues to the ovaries [6]. Therefore, the accumulation and transport of essential nutrients are key research topics in broodstock nutrition.

Lipids and their constituent fatty acids are major organic components in fish, serving numerous physiological roles, including membrane structure, cellular signaling, and energy storage. In teleosts, reproduction demands substantial lipid-derived energy, making reproductive success and offspring survival closely dependent on lipid availability and quality during gametogenesis [7]. At the cellular level, lipid droplets function as intracellular organelles responsible for lipid storage and fatty acid mobilization through interactions with other organelles and cytosolic proteins [8]. Fish lipids are particularly rich in essential long-chain polyunsaturated fatty acids (LC-PUFAs), which are acquired through diet and play critical roles in physiological processes [9]. Among LC-PUFA, eicosapentaenoic acid (20:5n-3, EPA) and docosahexaenoic acid (22:6n-3, DHA) from the n-3 LC-PUFA family, as well as arachidonic acid (20:4n-6, ARA) from the n-6 LC-PUFA family, have been proven to improve fecundity, egg hatching and viability, and larval survival at optimal concentrations and ratios in the gonads [10,11]. However, the composition of these essential fatty acids varies markedly among individuals and tissues [12,13] and is dynamically influenced by seasonal changes [14,15,16], dietary shifts [12,17,18], and physiological processes, particularly those associated with reproduction [19,20].

Spotted scat (*Scatophagus argus*) is an Indo-Pacific species of considerable economic importance, as it is a popular ornamental fish while also serving as a premium food fish in Asian markets [21,22]. This species typically reaches sexual maturity at 2–3 years of age, with its growth and gonadal development being highly environment-dependent. Optimal maturation occurs at salinities of 5–15 ppt and temperatures of 25–30 °C, typically reaching a spawning peak in July [23,24]. However, with the increasing market demand, the growing shortage of seed supply has hindered the further development of the industry. The stability of seed supply depends heavily on high-quality egg production, which is fundamentally determined by the nutritional status of the oocytes [25]. Previous studies on captive female *S. argus* have shown that ovarian maturation and regression are accompanied by annual fluctuations in reproductive hormones, indicating that ovarian development is regulated by endogenous physiological mechanisms [26]. Recent transcriptomic analyses of both the liver and ovary in adult female *S. argus* have demonstrated that dietary fish oil supplementation significantly regulates genes involved in hormone, lipid, and glucose metabolism, thereby effectively promoting ovarian development [27,28]. These studies reveal the dependence of gonadal development on lipids and also demonstrate the coordinated metabolic interaction between the liver and ovary. However, despite the critical role of hepatic–ovarian fatty acid trafficking in supporting vitellogenesis, specific information on the seasonal dynamics of fatty acid profiles in the liver and ovary remains unreported for *S. argus*. We hypothesized that lipid droplet accumulation and fatty acid composition would follow distinct stage-specific patterns, with ovarian lipid peaks occurring during oocyte maturation, and hepatic lipid accumulation serving as energy reserves prior to reproduction. Therefore, the present study monitored the fatty acid composition and lipid droplet accumulation in the liver and ovary of captive *S. argus* broodstock the course of one year, aiming to identify the critical periods of lipid mobilization and accumulation. These findings will provide valuable insights into oocyte quality and reproductive success in *S. argus*, and offer useful information for the development of optimized broodstock diets in aquaculture.

## 2. Materials and Methods

### 2.1. Experimental Fish and Rearing Conditions

The experiment was conducted at the Marine Biological Research Base of Guangdong Ocean University, using 2-year-old female fish (reared continuously at the base since the juvenile stage). Based on morphological differences between males and females, 120 females were selected and cultured in an outdoor open-air cement pond (12 m × 5 m × 2 m). The experimental water (salinity 8‰, a seawater–freshwater mix) was prepared and maintained in dedicated storage tanks. In these tanks, the water was disinfected with 1.0 mg/L chlorine dioxide and pre-aerated to ensure stability before use. Salinity was monitored weekly using a handheld refractometer and adjusted only when necessary to maintain consistent conditions throughout the study. To maintain stable dissolved oxygen, continuous aeration was applied to the rearing system during the entire trial. Dissolved oxygen levels ranged from 6.0 to 8.0 mg L^−1^, and pH was maintained at 7.5–8.5. To maintain water quality, a daily water exchange of 15% was performed. The commercial feed contained 44% crude protein and 12% crude fat. The fish were fed twice daily using feeding trays, with each feeding amounting to 1.5–2.5% of their total body weight, and adjusted dynamically based on their daily feeding response. During the winter months, the feeding frequency was reduced to once daily, and the feeding amount per meal was correspondingly decreased to approximately 0.5–1.0% of body weight, depending on their appetite.

### 2.2. Sample Collection

Samples were collected every two months from May 2019 to March 2020, with water temperature recorded at each sampling event (May 2019, 25.5 °C; July, 29.8 °C; September, 28.2 °C; November, 25.3 °C; January 2020, 20.7 °C; and March, 22.5 °C); the number of fish sampled per month was March (*n* = 6), May (*n* = 15), July (*n* = 8), September (*n* = 5), November (*n* = 4), and January (*n* = 5). Bi-monthly sampling was adopted to minimize repeated handling stress on broodstock females during the anticipated reproductive period, thereby protecting reproductive success and animal welfare. Immediately after the key sampling in July, artificial propagation was carried out via hormone-induced spawning. Therefore, the data measured in July were used as reference values for various indicators during the ovarian maturation stage. Feeding was stopped 24 h before each sampling. Each fish’s body weight, length, gut weight, liver weight, and gonad weight were all taken. The following formulae were used to determine condition factor (CF), viscerosomatic index (VSI), hepatosomatic index (HSI), and gonadosomatic index (GSI) [29,30]:

CF = body weight (g)/body length (cm^3^) × 100%;
VSI (%) = visceral weight (g)/body weight (g) × 100%;
HSI (%) = liver weight (g)/body weight (g) × 100%;
GSI (%) = gonadal weight (g)/body weight (g) × 100%

Liver and ovary samples were snap-frozen in liquid nitrogen and stored at −80 °C for analysis of absolute fatty acid content and lipid droplet observation. Portions of ovarian tissues were also fixed in Bouin’s solution for histological examination. Ovarian development in spotted scat was classified into stages II, III, and IV according to the criteria described by Cui [31]. Notably, during the May sampling, ovaries at stages II, III, and IV were observed, and these samples were used to compare differences in fatty acid content across the distinct ovarian developmental stages at the same time point stages of ovarian development.

### 2.3. Histological Observation of Ovary

For each fish were collected and fixed for histological evaluation, ovarian tissues were fixed in Bouin’s solution for 24 h with gentle agitation. The fixed tissues were then dehydrated in a graded ethanol series: 75% ethanol (3 changes, 5–10 min each), 80% ethanol (1 change, 5 min), 90% ethanol (1 change, 5 min), 95% ethanol (1 change, 5 min), and 100% ethanol (3 changes, 5 min each). Subsequently, the samples were cleared by immersion in a xylene:ethanol mixture (1:1) for approximately 1 h, followed by two changes of pure xylene (xylene I and xylene II) for approximately 1 h each. Clearing time was adjusted based on tissue size and lipid content to achieve transparency without over-clearing. The cleared samples were infiltrated and embedded in paraffin: first in a xylene:paraffin mixture (1:1) for 30 min, followed by two changes of pure paraffin (paraffin I and paraffin II) for 30 min each. The paraffin-embedded tissues were sectioned at 6–8 μm thickness, stained with hematoxylin and eosin (H&E), and examined under a light microscope [32].

### 2.4. Oil Red O Staining of Ovary and Liver

Ovary and liver samples were fixed in 4% paraformaldehyde (PFA) overnight at 4 °C, followed by three washes (10 min each) in 1× phosphate-buffered saline containing 0.1% Tween-20 (PBST). The samples were then dehydrated in 30% sucrose solution overnight at 4 °C and embedded in optimal cutting temperature (OCT) compound. The OCT-embedded tissues were snap-frozen in isopentane pre-cooled with liquid nitrogen (or directly in liquid nitrogen). Cryosections were prepared, stained with Oil Red O, photographed under a light microscope, and analyzed for lipid droplet accumulation in the liver and ovary.

### 2.5. Determination of Absolute Fatty Acid Content in the Ovary and Liver

The absolute content of fatty acids in spotted scat liver and ovary samples was determined according to the National Food Safety Standard of the People’s Republic of China (GB 5009.168-2016 [33], Determination of Fatty Acids in Foods). Triglyceride undecanoate was used as the internal standard. Briefly, accurately weighed samples were subjected to acid hydrolysis with hydrochloric acid solution. The lipids were extracted with diethyl ether, followed by saponification with sodium hydroxide in methanol under alkaline conditions to produce fatty acid salts. Boron trifluoride-methanol was then added to convert the salts into fatty acid methyl esters (FAMEs). The FAMEs were dissolved in n-heptane, and a saturated sodium chloride solution was added to facilitate phase separation. The upper organic layer was collected, dried over anhydrous sodium sulfate, and transferred for analysis. A standard solution of FAMEs was injected into a gas chromatograph (Agilent 7890B, Agilent Technologies, Santa Clara, CA, USA) equipped with an SP-2560 capillary column (100 m × 0.25 mm × 0.25 μm) to identify chromatographic peaks. The sample FAME solutions were then injected, and fatty acid contents were quantified using the internal standard method based on peak areas and appropriate conversion factors.

### 2.6. Statistical Analysis Methods

All experimental data are presented as mean ± standard error (mean ± SE). Measurements were obtained from individual fish, with each fish considered an independent biological replicate. One-way analysis of variance (ANOVA) was performed using SPSS 17.0 software (SPSS Inc., Chicago, IL, USA), and differences were considered statistically significant at *p* < 0.05.

## 3. Results

### 3.1. Growth Profile of Spotted Scat Female Broodstock

From May onwards, body weight, total length, standard length, and condition factor (CF) of the female spotted scat broodstock showed a steady increase across the sampling months (Table 1). Gonadosomatic index (GSI) rose noticeably in July, with values remaining relatively stable in the other months. Hepatosomatic index (HSI) was clearly higher from November to March (winter and spring) than from May to September (summer and autumn). Viscerosomatic index (VSI) tended to be lower in May and September but higher in July and during the November–March period. While no statistically significant differences were found among months, the highest VSI value was recorded in March.

### 3.2. Ovarian Development

All ovarian samples from paraffin sections were examined under a light microscope, and their developmental stages were recorded. In May, approximately 40% of the ovaries had reached stage IV. By July, all ovaries (100%) were at stage IV. After September, ovarian regression occurred, with stage III predominating (accounting for 60%). In January, ovaries were mainly distributed between stages II and III, each representing 40%. By March, ovarian development progressed again, with stages III and IV. (Figures S1 and S2; Table 2).

### 3.3. Ovarian Lipid Droplet Accumulation

**Figure 1:**
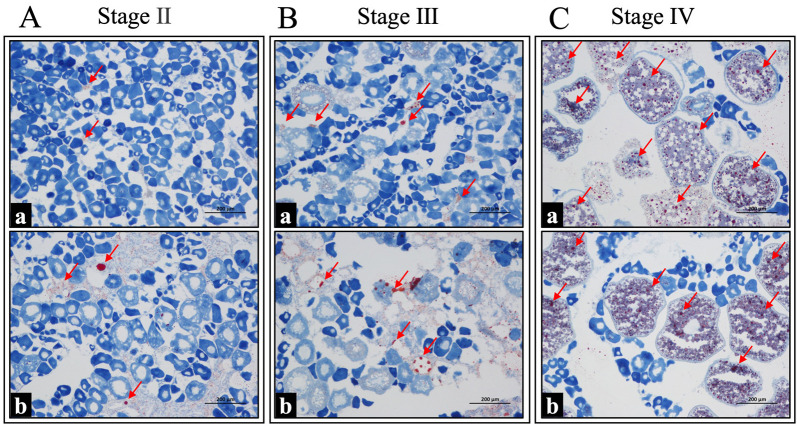
Oil Red O staining of ovarian lipid droplet accumulation in female spotted scat (*Scatophagus argus*) at different maturation stages: (**A**) stage II, (**B**) stage III, and (**C**) stage IV. Arrows indicate lipid droplets. Scale bar = 200 µm.

Lipid droplet accumulation in the ovaries of spotted scat broodstock was examined in cryosections stained with Oil Red O. In July, when all ovaries had reached stage IV, lipid droplets were most abundant. In contrast, accumulation remained low in November, when ovaries were predominantly at stages II and III, and this low level persisted into January. Overall, lipid droplet content clearly increased as ovarian development progressed from earlier to later stages (Figure 1). Representative Oil Red O-stained images of ovaries from different months are shown in Figures S3 and S4.

### 3.4. Liver Lipid Droplet Accumulation

**Figure 2:**
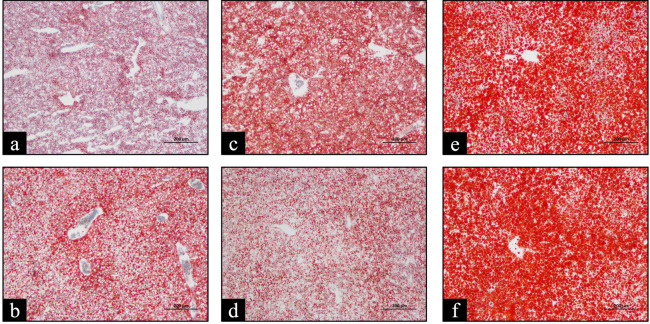
Representative Oil Red O-stained frozen sections of liver from female spotted scat (*Scatophagus argus*) broodstock at ovarian maturation stage IV, illustrating seasonal variation in lipid droplet accumulation. Panels show liver samples from (**a**) May (*n* = 9), (**b**) July (*n* = 6), (**c**) September (*n* = 5), (**d**) November (*n* = 4), (**e**) January (*n* = 5), and (**f**) March (*n* = 6). The lipid droplets are stained red. Scale bar = 200 µm.

Lipid droplet accumulation in the liver of female spotted scat broodstock was examined in cryosections. Accumulation was most pronounced in January (winter), followed by March (early spring), while the lowest levels were seen in May (pre-breeding) and July (during breeding) (Figure S5). Figure 2 shows representative images of liver tissue from stage IV females across the different months.

### 3.5. Absolute Fatty Acid Content in the Ovary

Seasonal variations in absolute fatty acid content were observed in the ovaries of spotted scat broodstock (Table 3). During the breeding period in July, levels of palmitic acid, stearic acid, oleic acid, γ-linoleic acid, linolenic acid, DHA, total SFA, MUFA, n-6 PUFA, and n-3 PUFA reached their highest values. ARA and EPA contents showed no significant differences across months. While most fatty acid levels in May were comparable to those in July, the n-3 PUFA/n-6 PUFA ratio was notably higher in May than in March.

### 3.6. Absolute Fatty Acid Content in the Liver

In the liver, absolute contents of SFA and ARA were noticeably lower in July compared to other months, with the highest levels recorded in January and March (Table 4). Contents of linoleic acid and total n-6 PUFA dropped markedly in May and July. Oleic acid levels were lower in May, July, and September than in January and March. Similarly, total MUFA was reduced in May, July, and September relative to the winter–spring period. EPA content and the n-3 PUFA/n-6 PUFA ratio stood out as significantly higher in May than in the other months. DHA and total n-3 PUFA also peaked in May, exceeding values seen in July, November, and March.

### 3.7. The Absolute Content of Fatty Acids in Ovary and Liver of Female Broodstock at Stage II–IV

Based on the sampling data from May ovarian, contents of palmitic acid, oleic acid, MUFA, gamma-linolenic acid, eicosatrienoic acid, docosadienoic acid, n-6 PUFA, EPA, DHA, n-3 PUFA were significantly higher in stage IV than at stages II and III (Table 5). SFA, linoleic acid, and arachidonic acid (ARA) levels in stage IV ovaries were higher than those in stage III and also exceeded stage II values (though the difference from stage II was not significant). Additionally, the n-3/n-6 PUFA ratio was markedly higher in stages III and IV than in stage II.

In the liver, absolute contents of SFA, MUFA, n-6 PUFA, and n-3 PUFA showed a gradual increase as ovarian development progressed from stage II to stage IV (Table 5). However, none of these differences reached statistical significance among the stages.

## 4. Discussion

### 4.1. Effect of Gonad Development on Fatty Acid and Lipid Droplets Contents

Fish sexual maturity depends primarily on age, while seasonal changes influence the stage of gonad development in sexually mature individuals [15,16]. The gonads of most fish progress through developmental stages I to V, significant variations were also observed in the fatty acid composition [34]. Fatty acids serve as structural components of cell membranes and precursors for prostaglandin synthesis; they bind to specific amino acid residues in proteins, anchoring these proteins to membranes. Beyond their role in mitochondrial β-oxidation for energy supply, fatty acids are stored as triglycerides in cellular lipid droplets and are enzymatically hydrolyzed into free fatty acids and glycerol when energy is required [35]. Lipid droplets are complex, metabolically active organelles that participate directly in transmembrane transport and phospholipid metabolism [36]. As ovarian cells mature, morphological changes in oocyte lipid droplets can serve as indicators of ovulation and maturation stages in Japanese eel oocytes [37].

During ovarian development in olive flounder (*Paralichthys olivaceus*) from stages III to V, DHA content in the liver remained stable, while ovarian DHA levels increased. In wild olive flounder broodstock, the liver DHA/EPA ratio was higher at stages III–IV than at stage V, suggesting preferential mobilization of n-3 LC-PUFA from the liver to support ovarian development and selective accumulation in the ovary [38,39]. In silver carp broodstock, ovary crude fat content increased initially and then declined, while liver fat gradually decreased. Both ovary and liver fatty acids were primarily MUFAs, with DHA being the main LC-PUFA [26]. Similarly, in *Odontobutis potamophila* broodstock, ovaries at stages III to V had higher concentrations of oleic acid, palmitic acid, palmitoleic acid, linoleic acid, EPA, and DHA compared to earlier stages [40].

In this study, fatty acid contents in the ovary of spotted scat varied significantly with ovarian development. Ovarian levels of SFA, MUFA, n-6 PUFA, n-3 PUFA, EPA, and DHA increased steadily from stage II to stage IV, indicating a selective accumulation as the ovary matured. These findings highlight the crucial role of ovarian development in lipid deposition and fatty acid composition in spotted scat. It is noteworthy that hepatic fatty acid content showed an increasing trend with advancing ovarian developmental stages in May, although no significant differences were observed among the indicators. This phenomenon warrants further mechanistic investigation.

### 4.2. Seasonal Effect on Broodstock Ovarian Development in View of FAs and Lipid Droplets

Seasonal changes influence the physiology, fatty acid profiles, and lipid droplet dynamics in fish [41,42]. In the present study, ovarian lipid droplet accumulation in spotted scat peaked in July, coinciding with ovarian maturation at stage IV. During this period, the GSI reached its highest value, and the contents of major fatty acid groups (SFA, MUFA, n-6 PUFA, n-3 PUFA) along with DHA were significantly elevated, indicating the mobilization of these fatty acids to support ovarian development.

This observation is consistent with patterns reported in other teleost species. In pikeperch (*Sander lucioperca*), lipid droplets (LDs) serve as a primary morphological indicator, first appearing in the early vitellogenic phase and reaching maximum abundance at the final maturation stage [43]. Moreover, LD fragmentation together with the cortical reaction has been demonstrated to act as dual predictors of egg quality [44]. Similarly, in Eurasian perch (*Perca fluviatilis*), dynamic changes in oocyte LD composition and morphology have been reported during induced spawning, with progressive accumulation occurring as ovarian development advances [45]. These findings indicate that LD accumulation is closely associated with vitellogenesis and final oocyte maturation in teleost fishes.

In comparison, similar seasonal patterns were reported in other species. In white sea bream (*Diplodus sargus*), ovarian lipid accumulation occurred from pre-spawning (e.g., November) to peak maturation, with a more pronounced increase in neutral lipids than in polar lipids [46]. For Senegalese sole (*Solea senegalensis*), the optimal dietary ARA requirement reached 3.9% of total fatty acids in summer and early autumn, compared to only 2.2% in winter (average 3.0% year-round) [47]. In Chondrostoma regium, with the highest lipid levels in January (pre-spawning/winter) and significant increases in polyunsaturated fatty acids (PUFAs), including DHA and EPA, by April during peak maturation and spawning [48].Similarly, in northern pike (*Esox lucius*), as ovaries recrudesced from September to January (pre-spawning to maturation), there were notable shifts in ovarian and liver fatty acid composition, including decreases in certain PUFAs in liver polar lipids accompanied by selective accumulation and retention of essential fatty acids like DHA in ovarian tissue to support gonadal growth and reproductive demands during colder seasons [49]. In common carp, PUFAs, especially DHA and ARA, were crucial for ovarian maturation, with ovarian lipid content positively correlated with GSI [50,51]. Therefore, during ovarian development, lipid mobilization and fatty acid profiles are tightly regulated. While seasonal factors can influence gonadal activity, the stage-specific changes in lipid composition may be the primary drivers of reproductive success [52].

Interestingly, despite a marked post-spawning decline in GSI, body weight and body length continued to increase from July to September, indicating a shift in energy allocation from gonadal development toward somatic growth and tissue recovery. Such sequential energy partitioning, characterized by reproductive investment followed by compensatory somatic growth, represents a common strategy in iteroparous teleosts [53,54]. Subsequently, GSI remained low from September through spring, likely reflecting reduced gonadal activity associated with seasonal declines in feeding and temperature.

### 4.3. Changes of Liver Lipid Droplets and Fatty Acids in Female Broodstock

Anorexia is common in many fish species before spawning, when endogenous nutrients are mobilized and transformed to support the final stages of gonadal development [55]. In red drum (*Sciaenops ocellatus*), active feeding occurs in spring and summer, followed by spawning in autumn; the liver serves as the primary lipid storage site, with stored fat rapidly mobilized for reproduction within a month and large amounts of intraperitoneal lipids consumed in late summer [56]. In *Leptoclinus maculatus*, neutral lipids are transferred from the liver to the ovary from summer to autumn, with both tissues accumulating considerable lipids as the ovary matures in autumn [57]. Hepatocytes in wild trout during early vitellogenesis are completely filled with lipid droplets, and from late yolk formation through post-oviposition, the amount of lipid in the cells steadily declines [58].

In this study, the VSI increased significantly from winter to spring, with substantial mesenteric fat and hepatic lipid droplets (LDs) observed in Jan, identifying the liver and abdominal cavity as primary lipid reservoirs. As maturation progressed (March–May), hepatic DHA and total n-3 PUFA increased, peaking the n-3/n-6 ratio in May. During the reproductive peak (May–July), the synchronized depletion of hepatic LDs, SFA, MUFA, and n-6 PUFA coincided with a rapid GSI increase and ovarian lipid enrichment. This suggests an extensive lipid transfer from the liver to developing oocytes to meet the high energetic demands of maturation. However, the lack of continuous water temperature monitoring in this study limits a precise correlation between these metabolic shifts and fine-scale environmental thermal fluctuations.

## 5. Conclusions

In summary, the liver of spotted scat accumulates lipids during winter and spring to store energy for ovarian development. Before the breeding season, the liver is responsible for absorbing fatty acids, such as n-3 LC-PUFA, and transferring them to the ovary. Ovarian development in *S. argus* may drive the annual variations in lipid droplet accumulation within both the liver and ovary, though the underlying physiological mechanisms remain to be fully elucidated. Future studies integrating controlled thermal regimes, higher-frequency sampling, and multi-tissue lipid profiling are warranted to refine nutritional protocols and optimize broodstock management.

## Figures and Tables

**Table 1:** Seasonal changes in body weight, total length, standard length, and condition factor (CF) of female spotted scat (*Scatophagus argus*) from May 2019 to March 2020.

Year	Month (Sampling Size)	Weight (g)	Total Length (cm)	Standard Length (cm)	Condition Factor (CF)	Gonadosomatic Index (GSI)	Hepatosomatic Index (HIS)	Viscerosomatic Index (VSI)
2019	May (*n* = 15)	221.66 ± 7.34 ^e^	20.11 ± 0.23 ^c^	17.35 ± 0.19 ^c^	4.23 ± 0.06 ^c^	1.96 ± 0.31 ^b^	1.60 ± 0.09 ^b^	10.29 ± 0.32 ^b^
2019	July (*n* = 8)	253.97 ± 8.12 d^e^	20.59 ± 0.21 ^c^	17.66 ± 0.15 ^c^	4.61 ± 0.11 ^c^	8.10 ± 1.34 ^a^	2.29 ± 0.14 ^b^	15.25 ± 1.23 ^a^
2019	September (*n* = 5)	310.77 ± 21.41 ^cd^	21.78 ± 0.40 ^b^	18.70 ± 0.26 ^b^	4.72 ± 0.15 ^bc^	1.37 ± 0.19 ^b^	2.05 ± 0.30 ^b^	8.10 ± 0.67 ^b^
2019	November (*n* = 4)	375.76 ± 15.58 ^bc^	22.16 ± 0.32 ^b^	19.28 ± 0.28 ^b^	5.25 ± 0.23 ^ab^	1.93 ± 0.97 ^b^	3.67 ± 0.51 ^a^	14.10 ± 1.68 ^a^
2020	January (*n* = 5)	385.54 ± 24.01 ^b^	22.38 ± 0.53 ^b^	19.47 ± 0.47 ^b^	5.21 ± 0.17 ^ab^	1.72 ± 0.57 ^b^	4.17 ± 0.42 ^a^	15.56 ± 0.75 ^a^
2020	March (*n* = 6)	502.51 ± 22.00 ^a^	23.89 ± 0.17 ^a^	20.89 ± 0.15 ^a^	5.51 ± 0.19 ^a^	2.78 ± 1.00 ^b^	4.05 ± 0.53 ^a^	17.86 ± 1.19 ^a^

The data in the table are represented by “mean ± standard error”, and different lowercase letters indicated significant differences (*p* < 0.05).

**Table 2:** Seasonal distribution of ovarian maturation stages (I–IV) in female spotted scat (*Scatophagus argus*) sampled bi-monthly from May 2019 to March 2020.

Gonad Development Stage	May	July	September	November	January	March
I	0	0	0	0	0	0
II	5 (33.3)	0	0	1 (25%)	2 (40%)	0
III	4 (26.7)	0	3 (60%)	2 (50%)	2 (40%)	3 (50%)
IV	6 (40%)	8 (100%)	2 (40%)	1 (25%)	1 (20%)	3 (50%)
V	0	0	0	0	0	0
Sampling size (*n*)	*n* = 15	*n* = 8	*n* = 5	*n* = 4	*n* = 5	*n* = 6

**Table 3:** Ovarian fatty acid content in female spotted scat (*Scatophagus argus*) sampled bi-monthly from May 2019 to March 2020(g/100 g).

Fatty Acid/Month/Sample Size	May (*n* = 9)	July (*n* = 8)	September (*n* = 5)	November (*n* = 4)	January (*n* = 5)	March (*n* = 6)
C14:0	0.053 ± 0.009 ^ab^	0.061 ± 0.005 ^a^	0.032 ± 0.011 ^ab^	0.023 ± 0.011 ^b^	0.022 ± 0.006 ^b^	0.036 ± 0.008 ^ab^
C15:0	0.011 ± 0.001 ^a^	0.012 ± 0.001 ^a^	0.008 ± 0.002 ^ab^	0.005 ± 0.001 ^b^	0.005 ± 0.001 ^b^	0.005 ± 0.000 ^b^
C16:0	0.734 ± 0.145 ^b^	1.350 ± 0.071 ^a^	0.524 ± 0.170 ^b^	0.464 ± 0.209 ^b^	0.512 ± 0.131 ^b^	0.715 ± 0.097 ^b^
C17:0	0.012 ± 0.002 ^ab^	0.014 ± 0.001 ^a^	0.010 ± 0.003 ^ab^	0.005 ± 0.002 ^ab^	0.005 ± 0.001 ^b^	0.006 ± 0.002 ^ab^
C18:0	0.168 ± 0.032 ^ab^	0.299 ± 0.016 ^a^	0.153 ± 0.044 ^b^	0.145 ± 0.049 ^b^	0.167 ± 0.036 ^ab^	0.211 ± 0.025 ^ab^
C20:0	ND ^1^	ND	ND	ND	0.004 ± 0.001	0.009 ± 0.003
C22:0	ND	ND	ND	ND	ND	ND
C24:0	0.014 ± 0.003	0.016 ± 0.002	ND	ND	ND	ND
SFA ^2^	0.989 ± 0.191 ^ab^	1.757 ± 0.090 ^a^	0.737 ± 0.233 ^b^	0.641 ± 0.270 ^b^	0.715 ± 0.176 ^b^	0.982 ± 0.127 ^ab^
C16:1n7	0.206 ± 0.070	0.302 ± 0.029	0.124 ± 0.051	0.082 ± 0.055	0.120 ± 0.052	0.209 ± 0.061
C18:1n9c	1.020 ± 0.313 ^b^	3.043 ± 0.359 ^a^	1.025 ± 0.418 ^b^	0.781 ± 0.514 ^b^	0.917 ± 0.351 ^b^	1.349 ± 0.338 ^b^
C20:1	0.061 ± 0.012	0.136 ± 0.012	0.060 ± 0.023	0.051 ± 0.027	0.077 ± 0.026	0.119 ± 0.026
C22:1n9	0.026 ± 0.016	0.015 ± 0.001	0.010 ± 0.002	0.008 ± 0.002	0.010 ± 0.001	0.013 ± 0.002
C24:1n9	0.017 ± 0.002 ^b^	0.036 ± 0.002 ^a^	0.024 ± 0.005 ^ab^	0.018 ± 0.004 ^b^	0.021 ± 0.003 ^b^	0.021 ± 0.004 ^b^
MUFA ^3^	1.330 ± 0.393 ^b^	3.532 ± 0.394 ^a^	1.244 ± 0.496 ^b^	0.939 ± 0.601 ^b^	1.145 ± 0.428 ^b^	1.711 ± 0.425 ^ab^
C18:2n6c	0.405 ± 0.125 ^b^	2.291 ± 0.246 ^a^	0.790 ± 0.351 ^b^	0.748 ± 0.549 ^b^	0.640 ± 0.274 ^b^	1.189 ± 0.337 ^ab^
C18:3n6	0.078 ± 0.034 ^b^	0.460 ± 0.048 ^a^	0.165 ± 0.073 ^b^	0.133 ± 0.098 ^b^	0.144 ± 0.074 ^b^	0.208 ± 0.061 ^ab^
C20:2	0.039 ± 0.009 ^b^	0.163 ± 0.016 ^a^	0.074 ± 0.027 ^ab^	0.072 ± 0.040 ^ab^	0.085 ± 0.025 ^ab^	0.122 ± 0.023 ^ab^
C20:3n6	0.074 ± 0.024 ^b^	0.434 ± 0.024 ^ab^	0.218 ± 0.092 ^ab^	0.281 ± 0.174 ^ab^	0.348 ± 0.143 ^ab^	0.487 ± 0.118 ^a^
C20:4n6	0.108 ± 0.013	0.135 ± 0.009	0.085 ± 0.020	0.077 ± 0.013	0.137 ± 0.022	0.129 ± 0.014
C22:2n6	0.024 ± 0.009 ^b^	0.058 ± 0.006 ^a^	0.006 ± 0.001 ^b^	0.004 ± 0.002 ^b^	0.005 ± 0.001 ^b^	0.006 ± 0.001 ^b^
n-6PUFA ^4^	0.728 ± 0.209 ^b^	3.541 ± 0.315 ^a^	1.335 ± 0.561 ^b^	1.314 ± 0.871 ^b^	1.358 ± 0.534 ^b^	2.142 ± 0.540 ^ab^
C18:3n3	0.046 ± 0.012	0.128 ± 0.012	0.057 ± 0.021	0.055 ± 0.040	0.045 ± 0.018	0.085 ± 0.023
C20:3n3	0.013 ± 0.003 ^b^	0.037 ± 0.004 ^a^	0.018 ± 0.005 ^ab^	0.013 ± 0.008 ^b^	0.015 ± 0.005 ^b^	0.023 ± 0.005 ^ab^
C20:5n3	0.092 ± 0.029	0.093 ± 0.009	0.042 ± 0.017	0.018 ± 0.010	0.029 ± 0.011	0.042 ± 0.010
C22:6n3	0.900 ± 0.278 ^ab^	1.700 ± 0.231 ^a^	0.688 ± 0.215 ^ab^	0.566 ± 0.261 ^b^	0.661 ± 0.195 ^ab^	0.657 ± 0.163 ^ab^
n-3 PUFA ^5^	1.047 ± 0.316 ^ab^	1.958 ± 0.246 ^a^	0.804 ± 0.255 ^ab^	0.653 ± 0.297 ^b^	0.748 ± 0.220 ^ab^	0.807 ± 0.200 ^ab^
n-3 LC-PUFA ^6^	1.002 ± 0.310 ^ab^	1.830 ± 0.241 ^a^	0.748 ± 0.235 ^ab^	0.598 ± 0.272 ^b^	0.704 ± 0.206 ^ab^	0.722 ± 0.177 ^ab^
n-3PUFA/n-6PUFA ^7^	1.404 ± 0.139 ^a^	0.549 ± 0.037 ^ab^	0.797 ± 0.144 ^ab^	0.743 ± 0.388 ^ab^	0.816 ± 0.373 ^ab^	0.378 ± 0.007 ^b^

The data in the table are represented by “mean ± standard error”, and the superscripts of the data in the same row are marked with different lowercase letters, which indicated significant differences (*p* < 0.05). ^1^ ND: Not detectable. ^2^ SFA: saturated fatty acid. ^3^ MUFA: Monounsaturated fatty acid. ^4^ n-6 PUFAs: n-6 polyunsaturated fatty acids. ^5^ n-3 PUFAs: n-3 polyunsaturated fatty acids. ^6^ n-3 LC-PUFA: n-3 long-chain polyunsaturated fatty acid. ^7^ n-3 PUFA/n-6 PUFA: The ratio of n-3 polyunsaturated fatty acids to n-6 polyunsaturated fatty acids.

**Table 4:** Hepatic fatty acid content (mg/g tissue) in female spotted scat (*Scatophagus argus*) sampled bi-monthly from May 2019 to March 2020 (g/100 g).

Fatty Acid/Month/Sample Size	May (*n* = 9)	July (*n* = 8)	September (*n* = 5)	November (*n* = 4)	January (*n* = 5)	March (*n* = 6)
C14:0	0.179 ± 0.028 ^bc^	0.127 ± 0.022 ^c^	0.229 ± 0.031 ^bc^	0.278 ± 0.033 ^b^	0.416 ± 0.021 ^a^	0.415 ± 0.032 ^a^
C15:0	0.036 ± 0.007	0.021 ± 0.004	0.040 ± 0.007	0.024 ± 0.005	0.035 ± 0.003	0.023 ± 0.004
C16:0	3.138 ± 0.576 ^b^	2.870 ± 0.488 ^b^	4.958 ± 0.601 ^b^	6.110 ± 1.173 ^ab^	8.666 ± 0483 ^a^	8.763 ± 1.153 ^a^
C17:0	0.038 ± 0.007	0.023 ± 0.004	0.045 ± 0.007	0.029 ± 0.008	0.041 ± 0.003	0.030 ± 0.004
C18:0	0.508 ± 0.075 ^c^	0.439 ± 0.057 ^c^	0.917 ± 0.104 ^bc^	1.391 ± 0.352 ^ab^	1.660 ± 0.144 ^a^	1.866 ± 0.282 ^a^
C20:0	0.018 ± 0.002 ^cd^	0.016 ± 0.002 ^d^	0.033 ± 0.004 ^bcd^	0.057 ± 0.018 ^abc^	0.068 ± 0.013 ^ab^	0.076 ± 0.015 ^a^
C22:0	0.010 ± 0.001 ^b^	0.009 ± 0.001 ^b^	0.020 ± 0.002 ^ab^	0.021 ± 0.005 ^ab^	0.024 ± 0.004 ^a^	0.029 ± 0.004 ^a^
C24:0	0.021 ± 0.002 ^b^	0.010 ± 0.001 ^b^	0.030 ± 0.003 ^b^	0.043 ± 0.009 ^ab^	0.069 ± 0.011 ^a^	0.068 ± 0.016 ^a^
SFA ^1^	3.946 ± 0.690 ^bc^	3.508 ± 0.577 ^c^	6.268 ± 0.739 ^bc^	7.946 ± 1.598 ^ab^	10.981 ± 0.656 ^a^	11.265 ± 1.479 ^a^
C16:1n7	0.557 ± 0.100 ^bc^	0.305 ± 0.061 ^c^	0.718 ± 0.099 ^bc^	1.030 ± 0.120 ^b^	1.972 ± 0.105 ^a^	1.913 ± 0.257 ^a^
C18:1n9c	2.335 ± 0.413 ^c^	2.078 ± 0.376 ^c^	4.312 ± 0.502 ^c^	4.828 ± 0.944 ^bc^	9.158 ± 0.793 ^a^	7.785 ± 1.297 ^ab^
C20:1	0.312 ± 0.052 ^c^	0.268 ± 0.057 ^c^	0.634 ± 0.075 ^c^	1.180 ± 0.268 ^bc^	2.362 ± 0.405 ^a^	2.043 ± 0.405 ^ab^
C22:1n9	0.050 ± 0.006 ^c^	0.044 ± 0.008 ^c^	0.099 ± 0.013 ^c^	0.147 ± 0.031 ^bc^	0.268 ± 0.050 ^a^	0.264 ± 0.048 ^ab^
C24:1n9	0.049 ± 0.004 ^ab^	0.033 ± 0.005 ^b^	0.058 ± 0.004 ^ab^	0.054 ± 0.008 ^ab^	0.076 ± 0.006 ^a^	0.070 ± 0.013 ^a^
MUFA ^2^	3.303 ± 0.565 ^b^	2.727 ± 0.503 ^b^	5.801 ± 0.682 ^b^	7.238 ± 1.339 ^b^	13.836 ± 1.311 ^a^	12.075 ± 1.996 ^a^
C18:2n6c	0.679 ± 0.095 ^b^	0.570 ± 0.101 ^b^	1.235 ± 0.165 ^ab^	1.178 ± 0.328 ^ab^	1.938 ± 0.191 ^a^	1.532 ± 0.263 ^a^
C18:3n6	0.235 ± 0.066 ^c^	0.386 ± 0.063 ^bc^	0.676 ± 0.083 ^ab^	0.617 ± 0.206 ^abc^	0.805 ± 0.064 ^a^	0.671 ± 0.116 ^ab^
C20:2	0.124 ± 0.017 ^c^	0.158 ± 0.035 ^c^	0.364 ± 0.034 ^bc^	0.389 ± 0.108 ^bc^	0.845 ± 0.124 ^a^	0.603 ± 0.109 ^ab^
C20:3n6	0.161 ± 0.025 ^d^	0.273 ± 0.050 ^d^	0.743 ± 0.081 ^cd^	1.006 ± 0.211 ^bc^	1.972 ± 0.156 ^a^	1.502 ± 0.286 ^ab^
C20:4n6	0.080 ± 0.006 ^bc^	0.050 ± 0.005 ^c^	0.108 ± 0.007 ^ab^	0.089 ± 0.013 ^b^	0.133 ± 0.011 ^a^	0.105 ± 0.011 ^ab^
C22:2n6	0.038 ± 0.010 ^bc^	0.029 ± 0.006 ^c^	0.057 ± 0.006 ^bc^	0.058 ± 0.015 ^abc^	0.113 ± 0.021 ^a^	0.092 ± 0.016 ^ab^
n-6PUFA ^3^	1.317 ± 0.204 ^c^	1.466 ± 0.250 ^c^	3.183 ± 0.321 ^bc^	3.338 ± 0.835 ^bc^	5.806 ± 0.522 ^a^	4.505 ± 0.789 ^ab^
C18:3n3	0.078 ± 0.010 ^bc^	0.033 ± 0.004 ^c^	0.098 ± 0.014 ^bc^	0.131 ± 0.033 ^ab^	0.189 ± 0.017 ^a^	0.135 ± 0.023 ^ab^
C20:3n3	0.033 ± 0.005 ^bc^	0.018 ± 0.004 ^c^	0.052 ± 0.009 ^ab^	0.035 ± 0.011 ^bc^	0.073 ± 0.010 ^a^	0.044 ± 0.007 ^abc^
C20:5n3	0.175 ± 0.031 ^a^	0.038 ± 0.007 ^b^	0.073 ± 0.021 ^b^	0.037 ± 0.010 ^b^	0.046 ± 0.003 ^b^	0.032 ± 0.004 ^b^
C22:6n3	1.691 ± 0.244 ^a^	0.513 ± 0.081 ^b^	1.252 ± 0.160 ^ab^	0.629 ± 0.133 ^b^	0.978 ± 0.084 ^ab^	0.664 ± 0.077 ^b^
n-3PUFA ^4^	1.977 ± 0.287 ^a^	0.602 ± 0.093 ^b^	1.475 ± 0.196 ^ab^	0.833 ± 0.183 ^b^	1.285 ± 0.112 ^ab^	0.874 ± 0.105 ^b^
n-3LC-PUFA ^5^	1.899 ± 0.278 ^a^	0.569 ± 0.091 ^b^	1.377 ± 0.184 ^ab^	0.701 ± 0.153 ^b^	1.097 ± 0.096 ^ab^	0.739 ± 0.087 ^b^
n-3PUFA/n-6PUFA ^6^	1.537 ± 0.095 ^a^	0.426 ± 0.026 ^b^	0.461 ± 0.043 ^b^	0.260 ± 0.022 ^b^	0.223 ± 0.008 ^b^	0.209 ± 0.020 ^b^

The data in the table are represented by “mean ± standard error”, and the superscripts of the data in the same row are marked with different lowercase letters, which indicated significant differences (*p* < 0.05). ^1^ SFA: saturated fatty acid. ^2^ MUFA: Monounsaturated fatty acid. ^3^ n-6 PUFAs: n-6 polyunsaturated fatty acids. ^4^ n-3 PUFAs: n-3 polyunsaturated fatty acids. ^5^ n-3 LC-PUFA: n-3 long-chain polyunsaturated fatty acids. ^6^ n-3 PUFA/n-6 PUFA: The ratio of n-3 polyunsaturated fatty acid to n-6 polyunsaturated fatty acid.

**Table 5:** Absolute fatty acid content in the ovary and liver of female spotted scat (*Scatophagus argus*) broodstock at different ovarian maturation stages (II–IV) sampled in May 2019 (g/100 g, *n* = 3).

Ovaries				Liver			
Fatty Acid	Stage: II	Stage: III	Stage: IV	Faty Acid	Stage: II	Stage: III	Stage: IV
C14:0	0.048 ± 0.007	0.036 ± 0.014	0.074 ± 0.018	C14:0	0.129 ± 0.012	0.169 ± 0.042	0.241 ± 0.067
C15:0	0.009 ± 0.001	0.008 ± 0.002	0.015 ± 0.002	C15:0	0.024 ± 0.004	0.030 ± 0.008	0.054 ± 0.015
C16:0	0.496 ± 0.039 ^b^	0.487 ± 0.146 ^b^	1.219 ± 0.228 ^a^	C16:0	1.854 ± 0.510	3.283 ± 0.788	4.277 ± 1.271
C17:0	0.007 ± 0.000	0.008 ± 0.001	0.019 ± 0.003	C17:0	0.026 ± 0.004	0.033 ± 0.009	0.054 ± 0.016
C18:0	0.124 ± 0.003	0.109 ± 0.028	0.270 ± 0.061	C18:0	0.375 ± 0.093	0.547 ± 0.139	0.601 ± 0.157
C20:0	0.010 ± 0.002	ND ^1^	ND	C20:0	0.016 ± 0.005	0.021 ± 0.005	0.017 ± 0.003
C22:0	ND	ND	ND	C22:0	0.011 ± 0.003	0.011 ± 0.002	0.008 ± 0.001
C24:0	0.009 ± 0.002	0.009 ± 0.001	0.022 ± 0.004	C24:0	0.017 ± 0.004	0.019 ± 0.004	0.025 ± 0.005
SFA ^2^	0.698 ± 0.051 ^ab^	0.651 ± 0.194 ^b^	1.618 ± 0.315 ^a^	SFA ^1^	2.452 ± 0.631	4.113 ± 0.997	5.275 ± 1.522
C16:1n7	0.085 ± 0.012 ^b^	0.087 ± 0.033 ^b^	0.446 ± 0.119 ^a^	C16:1n7	0.318 ± 0.077	0.617 ± 0.153	0.737 ± 0.208
C18:1n9c	0.553 ± 0.142 ^b^	0.417 ± 0.122 ^b^	2.090 ± 0.527 ^a^	C18:1n9c	1.434 ± 0.458	2.493 ± 0.596	3.077 ± 0.889
C20:1	0.059 ± 0.017	0.033 ± 0.014	0.089 ± 0.020	C20:1	0.196 ± 0.085	0.408 ± 0.087	0.334 ± 0.080
C22:1n9	0.060 ± 0.048	0.008 ± 0.002	0.011 ± 0.001	C22:1n9	0.040 ± 0.013	0.059 ± 0.012	0.049 ± 0.006
C24:1n9	0.015 ± 0.001	0.014 ± 0.003	0.022 ± 0.004	C24:1n9	0.043 ± 0.008	0.051 ± 0.008	0.054 ± 0.008
MUFA ^3^	0.772 ± 0.201 ^b^	0.560 ± 0.173 ^b^	2.658 ± 0.667 ^a^	MUFA ^2^	2.031 ± 0.638	3.628 ± 0.854	4.251 ± 1.174
C18:2n6c	0.250 ± 0.063 ^ab^	0.150 ± 0.039 ^b^	0.816 ± 0.233 ^a^	C18:2n6c	0.531 ± 0.120	0.653 ± 0.120	0.854 ± 0.232
C18:3n6	0.019 ± 0.002 ^b^	0.022 ± 0.005 ^b^	0.193 ± 0.060 ^a^	C18:3n6	0.152 ± 0.024	0.150 ± 0.018	0.404 ± 0.171
C20:2	0.026 ± 0.002 ^ab^	0.022 ± 0.005 ^b^	0.069 ± 0.017 ^a^	C20:2	0.098 ± 0.022	0.120 ± 0.019	0.156 ± 0.041
C20:3n6	0.029 ± 0.002 ^b^	0.035 ± 0.007 ^b^	0.159 ± 0.038 ^a^	C20:3n6	0.116 ± 0.018	0.156 ± 0.031	0.211 ± 0.061
C20:4n6	0.076 ± 0.016 ^b^	0.098 ± 0.009 ^ab^	0.148 ± 0.020 ^a^	C20:4n6	0.078 ± 0.003	0.079 ± 0.012	0.081 ± 0.017
C22:2n6	0.007 ± 0.001 ^b^	0.008 ± 0.002 ^b^	0.055 ± 0.015 ^a^	C22:2n6	0.022 ± 0.011	0.049 ± 0.014	0.043 ± 0.027
n-6PUFA ^4^	0.408 ± 0.054 ^b^	0.334 ± 0.065 ^b^	1.440 ± 0.369 ^a^	n-6PUFA ^3^	0.996 ± 0.194	1.206 ± 0.206	1.750 ± 0.517
C18:3n3	0.053 ± 0.028	0.017 ± 0.006	0.067 ± 0.019	C18:3n3	0.061 ± 0.014	0.088 ± 0.021	0.085 ± 0.016
C20:3n3	0.006 ± 0.000	0.006 ± 0.001	0.023 ± 0.006	C20:3n3	0.026 ± 0.007	0.033 ± 0.009	0.041 ± 0.011
C20:5n3	0.034 ± 0.005 ^b^	0.047 ± 0.013 ^b^	0.194 ± 0.045 ^a^	C20:5n3	0.107 ± 0.022	0.195 ± 0.059	0.222 ± 0.062
C22:6n3	0.278 ± 0.042 ^b^	0.498 ± 0.111 ^b^	1.923 ± 0.339 ^a^	C22:6n3	1.192 ± 0.186	1.880 ± 0.479	2.000 ± 0.507
n-3PUFA ^5^	0.369 ± 0.028 ^b^	0.566 ± 0.131 ^b^	2.207 ± 0.404 ^a^	n-3PUFA ^4^	1.386 ± 0.227	2.196 ± 0.568	2.348 ± 0.588
n-3LC-PUFA ^6^	0.316 ± 0.047 ^b^	0.549 ± 0.126 ^b^	2.140 ± 0.387 ^a^	n-3LC-PUFA ^5^	1.326 ± 0.214	2.108 ± 0.547	2.263 ± 0.575
n-3PUFA/n-6PUFA ^7^	0.935 ± 0.137 ^b^	1.663 ± 0.150 ^a^	1.614 ± 0.158 ^a^	n-3PUFA/n-6PUFA ^6^	1.413 ± 0.075	1.761 ± 0.173	1.437 ± 0.188

The data in the table are represented by “mean ± standard error”, and the lower case superscripts indicated significant differences (*p* < 0.05). ^1^ ND: Not detectable. ^2^ SFA: saturated fatty acid. ^3^ MUFA: Monounsaturated fatty acid. ^4^ n-6 PUFAs: n-6 polyunsaturated fatty acids. ^5^ n-3 PUFAs: n-3 polyunsaturated fatty acids. ^6^ n-3 LC-PUFA: n-3 long-chain polyunsaturated fatty acid. ^7^ n-3 PUFA/n-6 PUFA: The ratio of n-3 polyunsaturated fatty acids to n-6 polyunsaturated fatty acids.

## Data Availability

The data presented in this study are available upon request from the corresponding author.

## Supplementary Materials

The following supporting information can be downloaded at: https://www.mdpi.com/article/10.3390/ani16050748/s1, Figure S1: Histological observation of the ovary in female spotted scat (*Scatophagus argus*) sampled in different months: (A) May (*n* = 15) and (B) July (*n* = 8). Numbers at the top right (e.g., M5-1, M7-1) and bottom left (II, III, and IV) indicate the sample numbers and gonadal developmental stages, respectively. Scale bars are indicated at the bottom right of each image. Figure S2: Histological observation of the ovary in female spotted scat (*Scatophagus argus*) sampled in different months: (A) September (*n* = 5), (B) November (*n* = 4), (C) January (*n* = 5), and (D) March (*n* = 6). Numbers at the top right (e.g., M9-1, M11-1) and bottom left (II, III, and IV) indicate the sample numbers and gonadal developmental stages, respectively. Scale bars are indicated at the bottom right of each image. Figure S3: Oil Red O staining of ovarian cryosections in female spotted scat (*Scatophagus argus*) sampled in different months: (A) May (*n* = 9), (B) July (*n* = 6), (C) September (*n* = 5), and (D) November (*n* = 4). Numbers at the top right (e.g., M5-1, M7-1) and bottom left (II, III, and IV) indicate the sample numbers and gonadal developmental stages, respectively. Arrows indicate lipid droplets. Scale bar = 200 µm. Figure S4: Oil Red O staining of ovarian cryosections in female spotted scat (*Scatophagus argus*) sampled in different months: (A) January (*n* = 5) and (B) March (*n* = 4). Numbers at the top right (e.g., M1-1, M3-1) and bottom left (II, III, and IV) indicate the sample numbers and gonadal developmental stages, respectively. Arrows indicate lipid droplets. Scale bar = 200 µm. Figure S5: Oil Red O staining of liver cryosections in female spotted scat *(Scatophagus argus*) sampled in different months: (A) May (*n* = 9), (B) July (*n* = 6), (C) September (*n* = 5), (D) November (*n* = 4), (E) January (*n* = 5), and (F) March (*n* = 6). Numbers at the top right (e.g., M5-1, M7-1) and bottom left (II, III, and IV) indicate the sample numbers and gonadal developmental stages, respectively. Lipid droplets are indicated by the red staining. Scale bar = 200 µm.
