# Supplementary material for: Developmental and Seasonal Changes in Lipid Droplets and Fatty Acid Composition in the Ovary and Liver of Female Spotted Scat (Scatophagus argus)
Source: Animals (Basel). 2026 Feb 27;16(5):748. doi: 10.3390/ani16050748 (PMC12984172; doi:10.3390/ani16050748)
Supplement: Supplementary file 1 [file animals-16-00748-s001.zip › Supplementary figure.pdf]

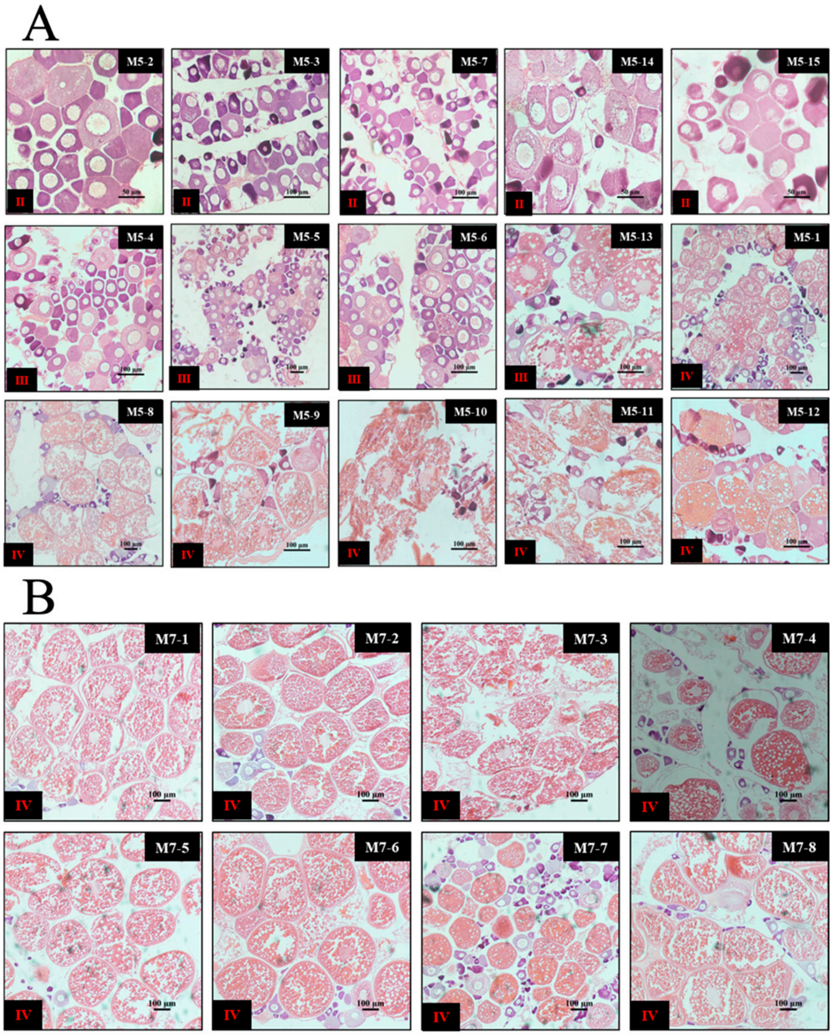

Figure S1. Histological observation of the ovary in female spotted scat (*Scatophagus argus*) sampled in different months: (A) May (n = 15) and (B) July (n = 8). Numbers at the top right (e.g., M5-1, M7-1) and bottom left (II, III, and IV) indicate the sample numbers and gonadal developmental stages, respectively. Scale bars are indicated at the bottom right of each image.

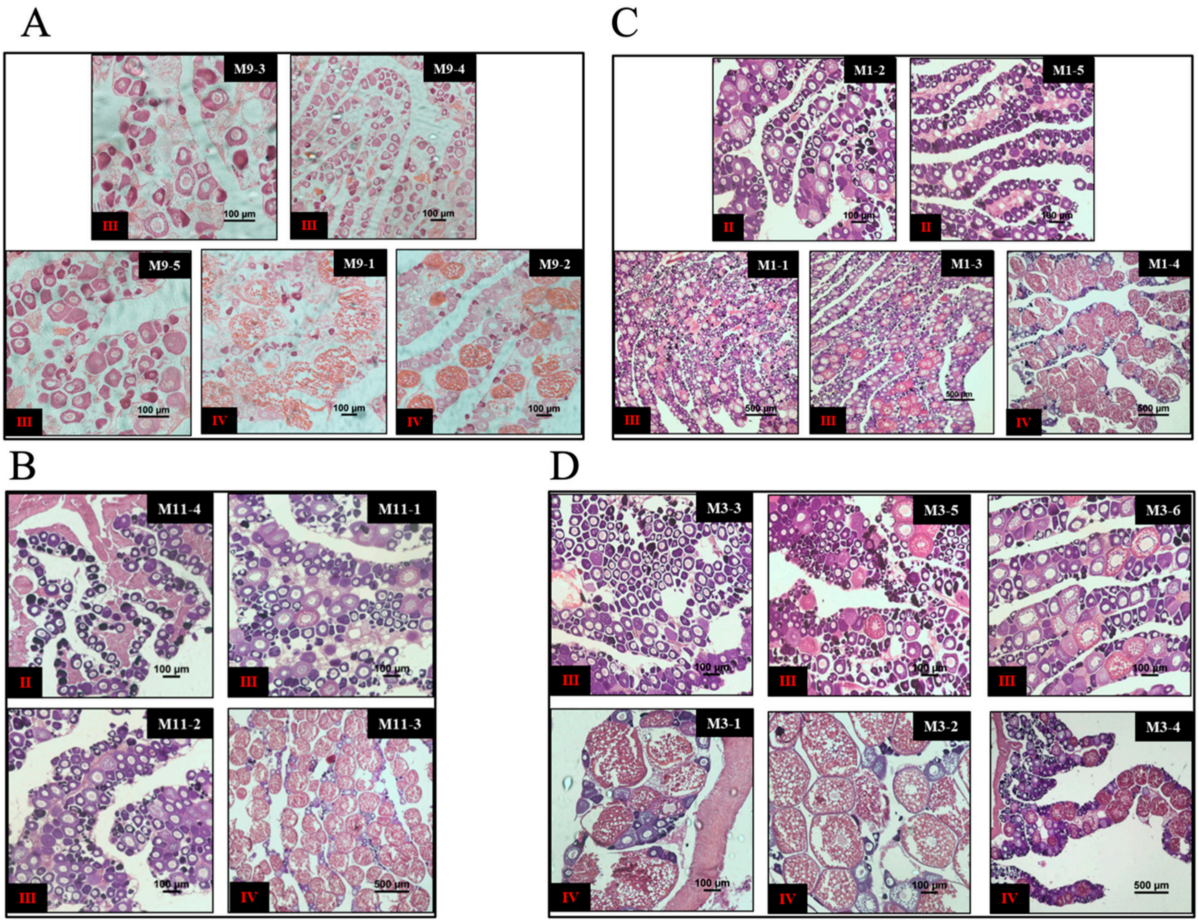

Figure S2. Histological observation of the ovary in female spotted scat (*Scatophagus argus*) sampled in different months: (A) September (n = 5), (B) November (n = 4), (C) January (n = 5), and (D) March (n = 6). Numbers at the top right (e.g., M9-1, M11-1) and bottom left (II, III, and IV) indicate the sample numbers and gonadal developmental stages, respectively. Scale bars are indicated at the bottom right of each image.

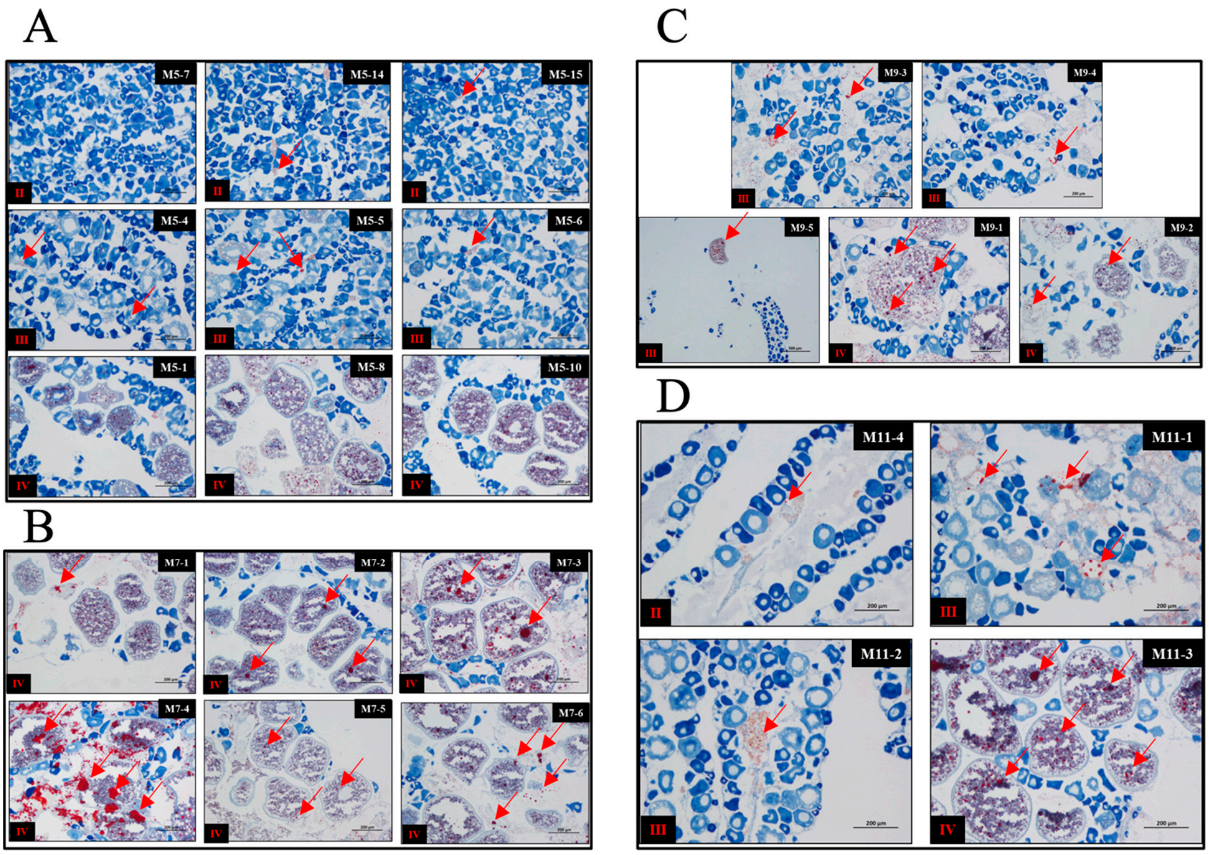

Figure S3. Oil Red O staining of ovarian cryosections in female spotted scat (*Scatophagus argus*) sampled in different months: (A) May (n = 9), (B) July (n = 6), (C) September (n = 5), and (D) November (n = 4). Numbers at the top right (e.g., M5-1, M7-1) and bottom left (II, III, and IV) indicate the sample numbers and gonadal developmental stages, respectively. Arrows indicate lipid droplets. Scale bar = 200 μm.

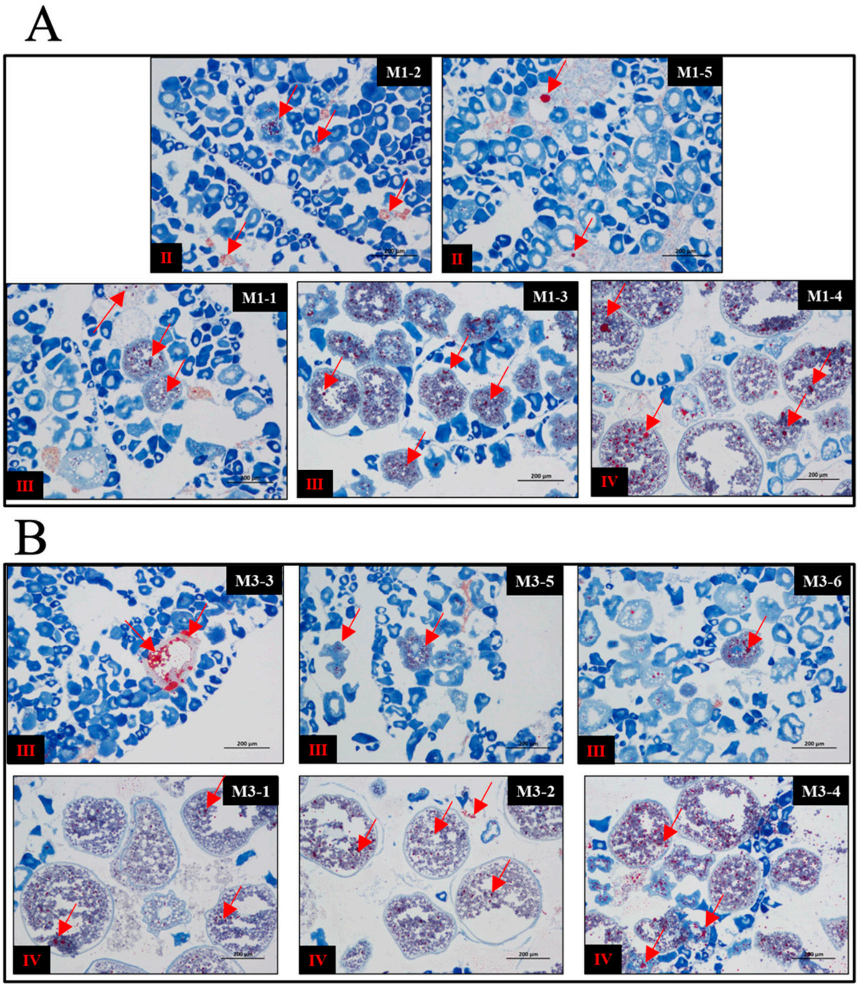

Figure S4. Oil Red O staining of ovarian cryosections in female spotted scat (*Scatophagus argus*) sampled in different months: (A) January (n = 5) and (B) March (n = 4). Numbers at the top right (e.g., M1-1, M3-1) and bottom left (II, III, and IV) indicate the sample numbers and gonadal developmental stages, respectively. Arrows indicate lipid droplets. Scale bar = 200 µm.

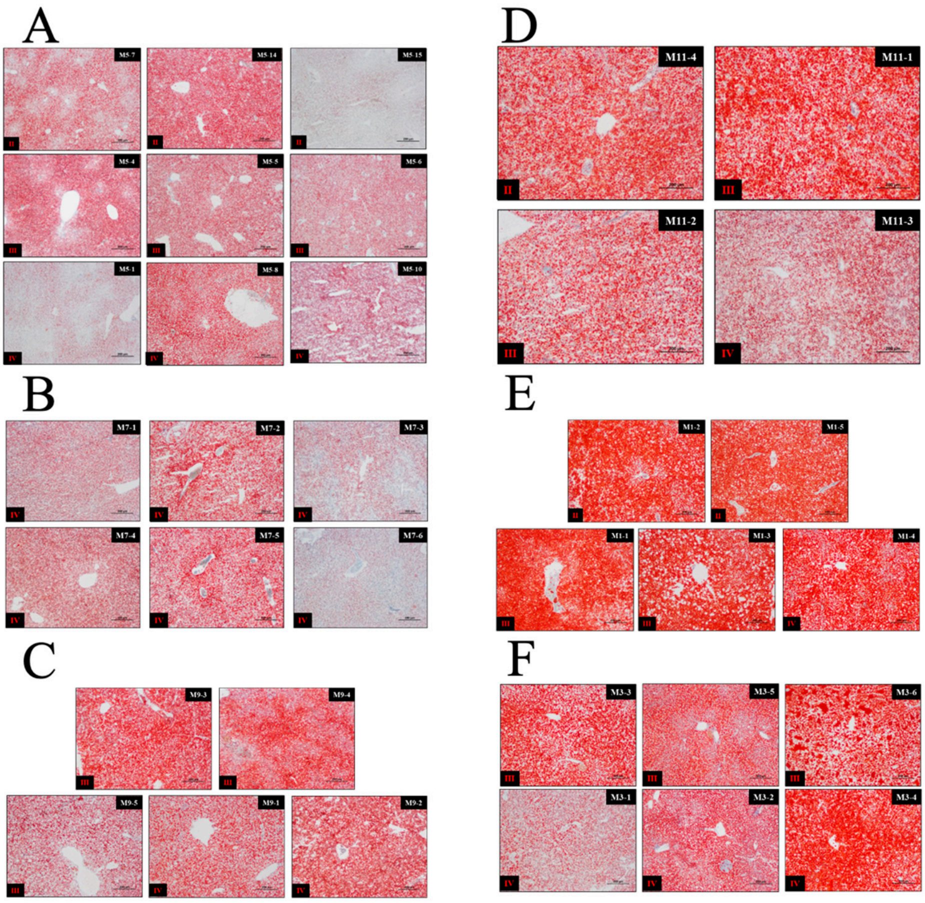

Figure S5. Oil Red O staining of liver cryosections in female spotted scat (*Scatophagus argus*) sampled in different months: (A) May (n = 9), (B) July (n = 6), (C) September (n = 5), (D) November (n = 4), (E) January (n = 5), and (F) March (n = 6). Numbers at the top right (e.g., M5-1, M7-1) and bottom left (II, III, and IV) indicate the sample numbers and gonadal developmental stages, respectively. Lipid droplets are indicated by the red staining. Scale bar = 200 µm.
