# Supplementary material for: Developmental and Seasonal Changes in Lipid Droplets and Fatty Acid Composition in the Ovary and Liver of Female Spotted Scat (Scatophagus argus)
Source: Animals (Basel). 2026 Feb 27;16(5):748. doi: 10.3390/ani16050748 (PMC12984172; doi:10.3390/ani16050748)
Supplement: Supplementary file 1 [file animals-16-00748-s001.zip › Supplementary File(s)/Supplementary figure.docx]

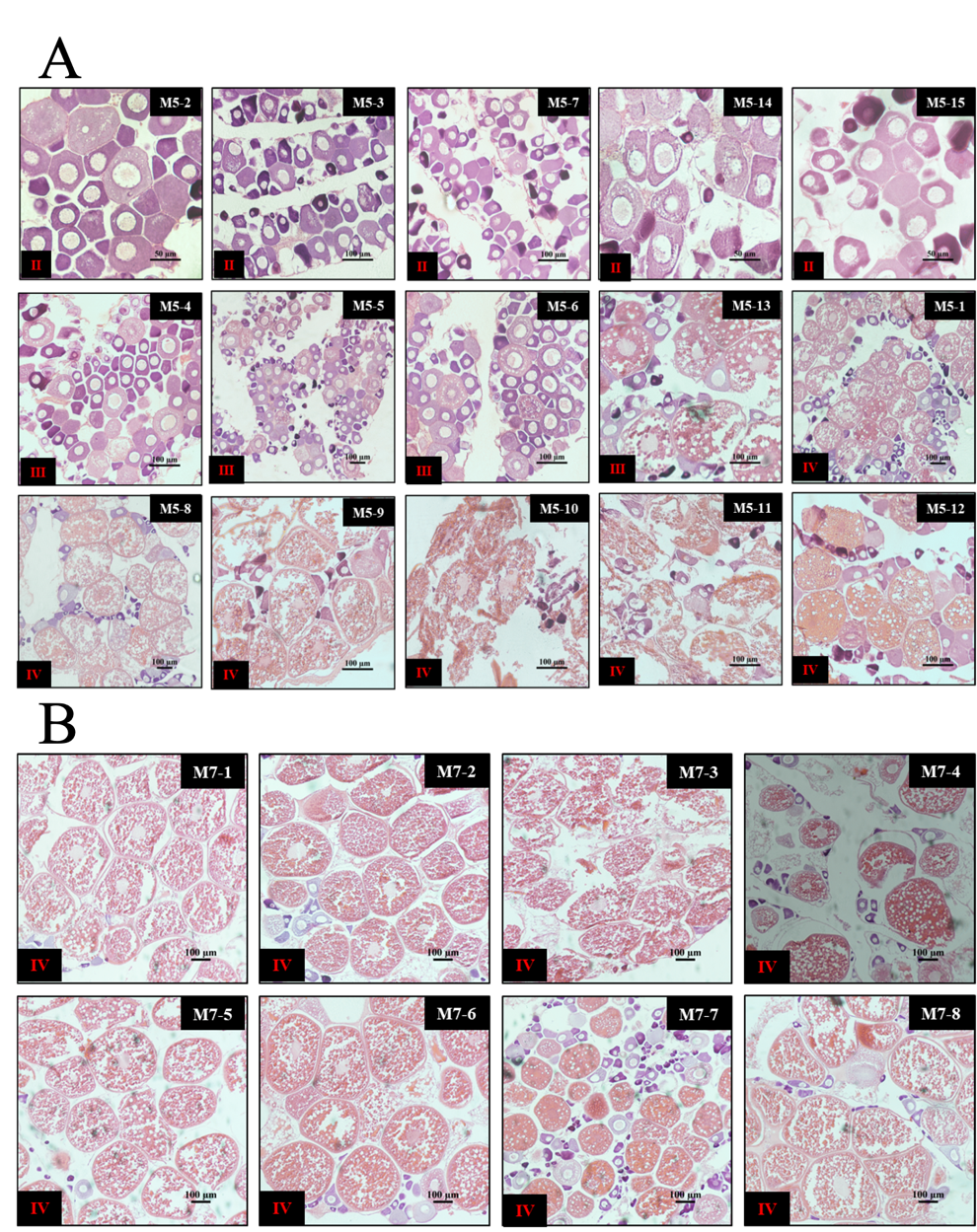


**Figure S1**. Histological observation of the ovary in female spotted scat (*Scatophagus argus*) sampled in different months: (A) May (n = 15) and (B) July (n = 8). Numbers at the top right (e.g., M5-1, M7-1) and bottom left (II, III, and IV) indicate the sample numbers and gonadal developmental stages, respectively. Scale bars are indicated at the bottom right of each image.

**
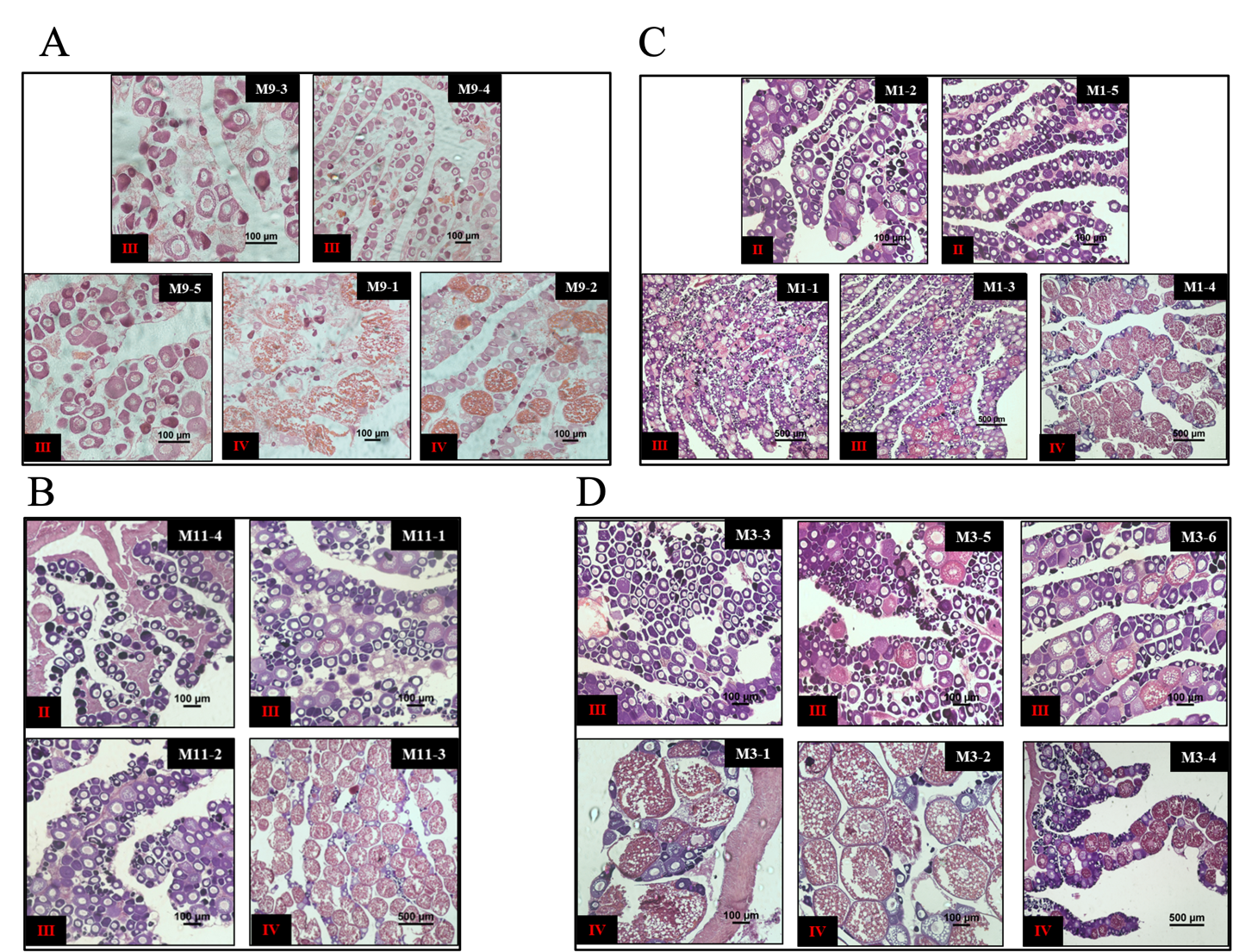
**

**Figure S2**. Histological observation of the ovary in female spotted scat (*Scatophagus argus*) sampled in different months: (A) September (n = 5), (B) November (n = 4), (C) January (n = 5), and (D) March (n = 6). Numbers at the top right (e.g., M9-1, M11-1) and bottom left (II, III, and IV) indicate the sample numbers and gonadal developmental stages, respectively. Scale bars are indicated at the bottom right of each image.


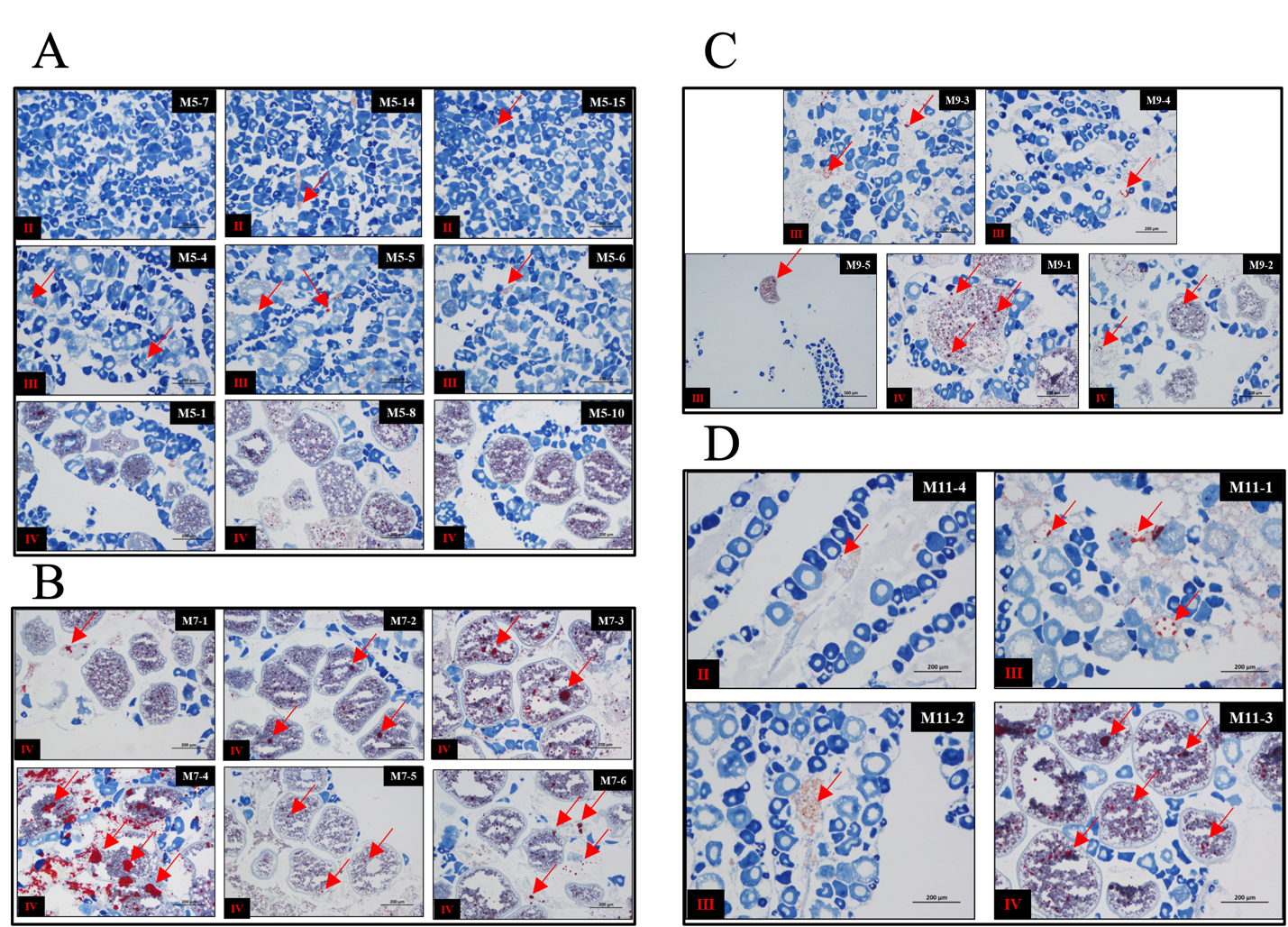


**Figure S3.** Oil Red O staining of ovarian cryosections in female spotted scat (*Scatophagus argus*) sampled in different months: (A) May (n = 9), (B) July (n = 6), (C) September (n = 5), and (D) November (n = 4). Numbers at the top right (e.g., M5-1, M7-1) and bottom left (II, III, and IV) indicate the sample numbers and gonadal developmental stages, respectively. Arrows indicate lipid droplets. Scale bar = 200 µm.


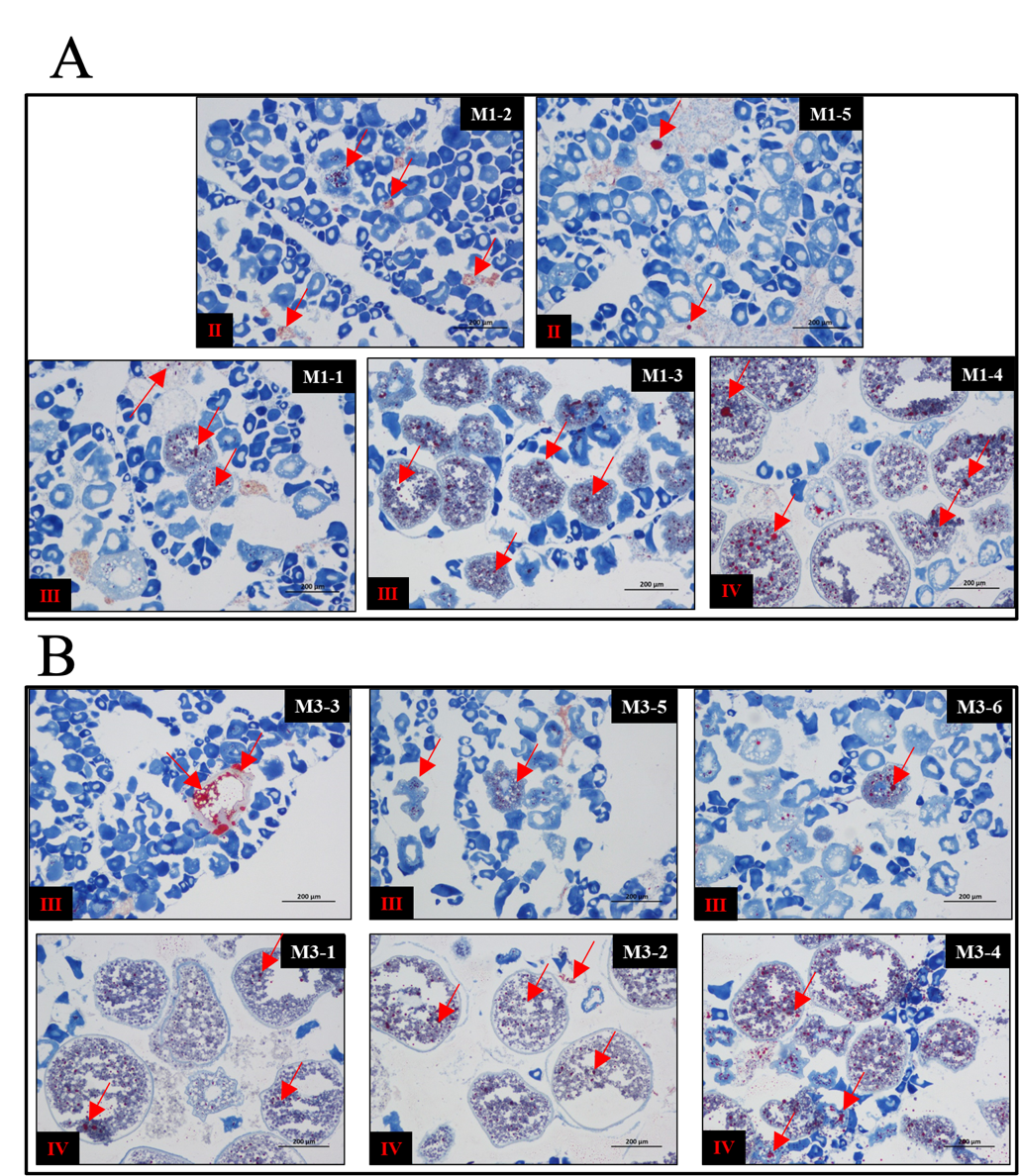


**Figure S4.** Oil Red O staining of ovarian cryosections in female spotted scat (*Scatophagus argus*) sampled in different months: (A) January (n = 5) and (B) March (n = 4). Numbers at the top right (e.g., M1-1, M3-1) and bottom left (II, III, and IV) indicate the sample numbers and gonadal developmental stages, respectively. Arrows indicate lipid droplets. Scale bar = 200 µm.
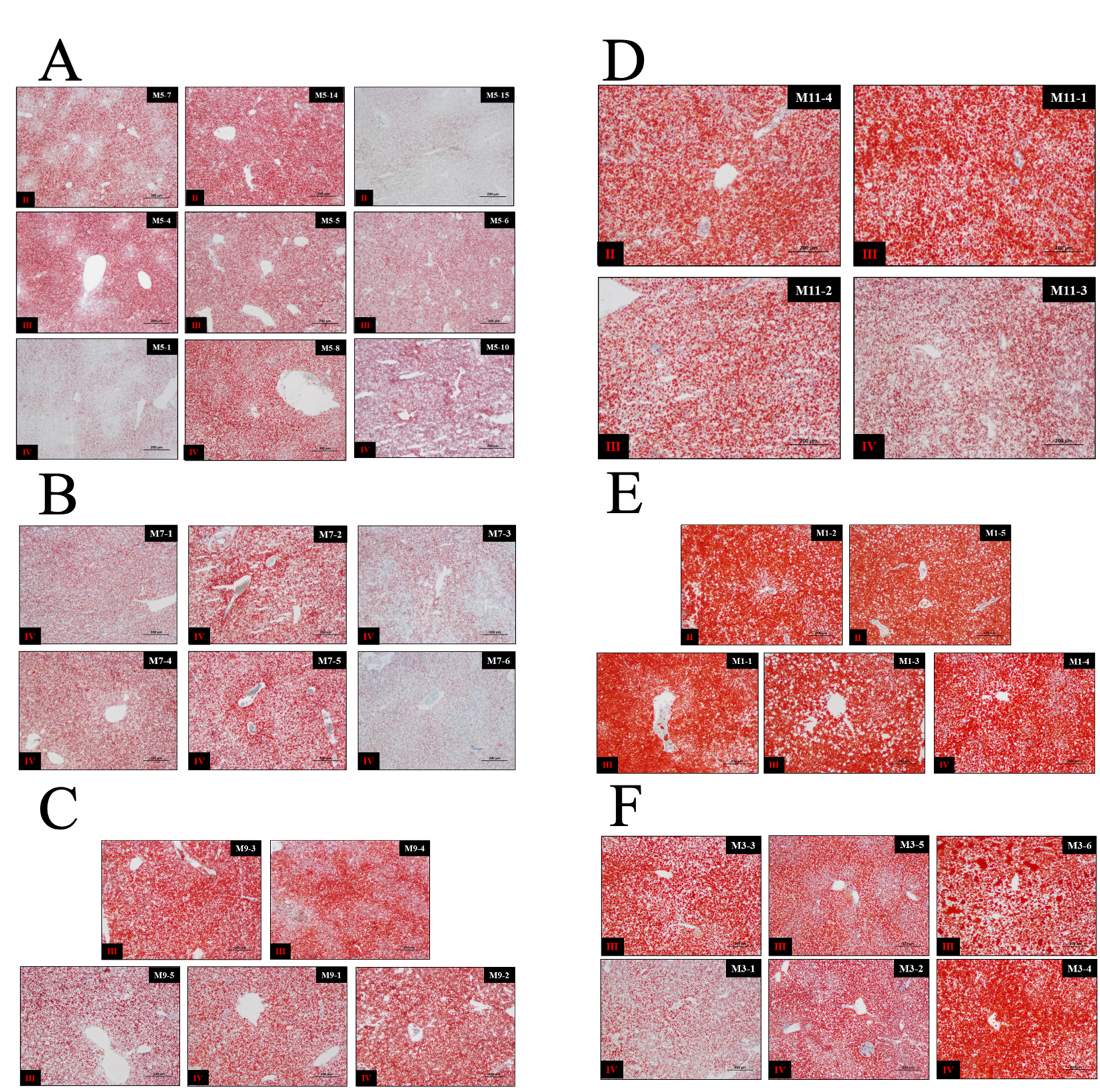


**Figure S5.** Oil Red O staining of liver cryosections in female spotted scat (*Scatophagus argus*) sampled in different months: (A) May (n = 9), (B) July (n = 6), (C) September (n = 5), (D) November (n = 4), (E) January (n = 5), and (F) March (n = 6). Numbers at the top right (e.g., M5-1, M7-1) and bottom left (II, III, and IV) indicate the sample numbers and gonadal developmental stages, respectively. Lipid droplets are indicated by the red staining. Scale bar = 200 µm.
