# Supplementary figures and images for: Developmental and Seasonal Changes in Lipid Droplets and Fatty Acid Composition in the Ovary and Liver of Female Spotted Scat (Scatophagus argus)
Source: Animals (Basel). 2026 Feb 27;16(5):748. doi: 10.3390/ani16050748 (PMC12984172; doi:10.3390/ani16050748)

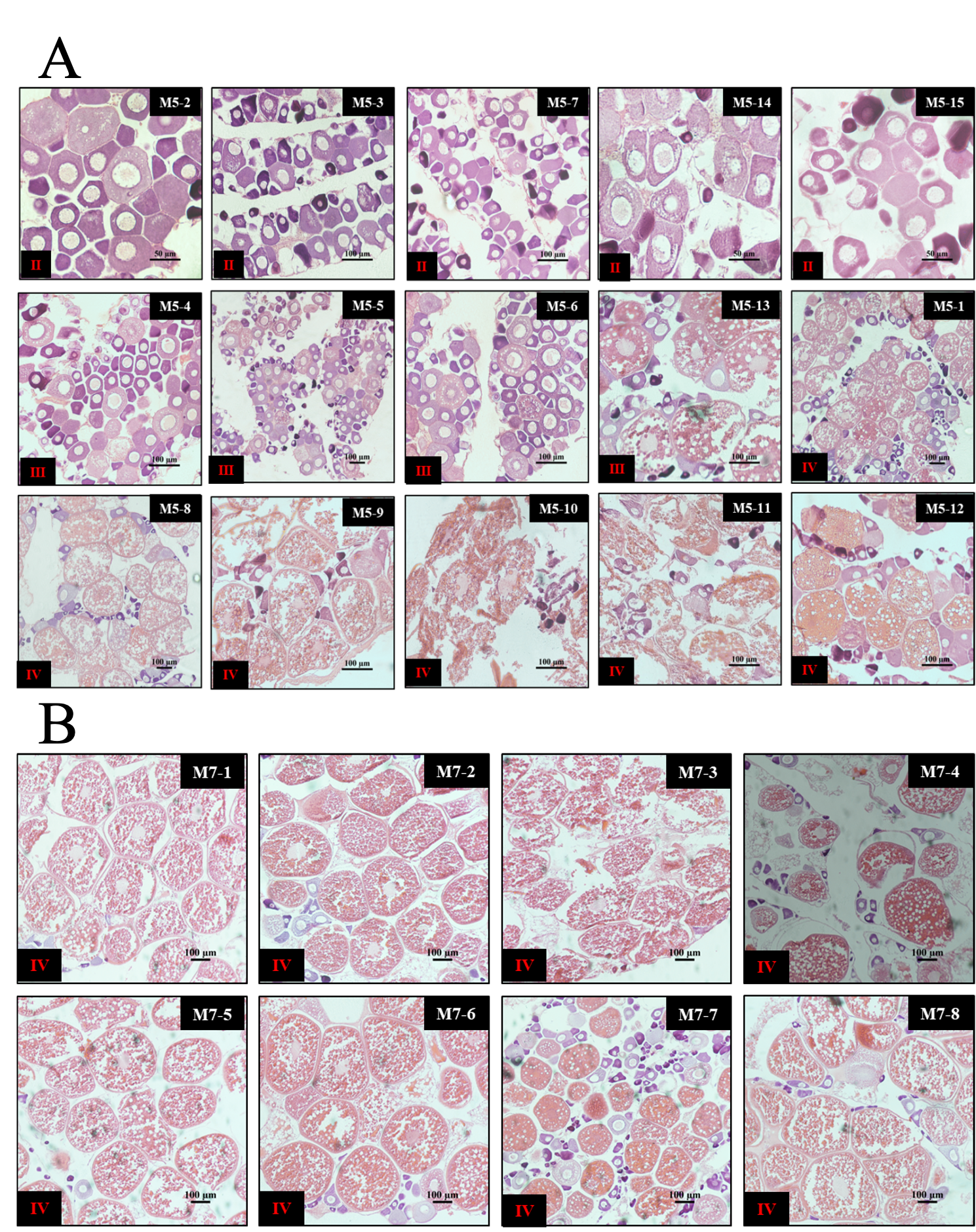

Supplement: Supplementary file 1 [file animals-16-00748-s001.zip › Figure S1.png]

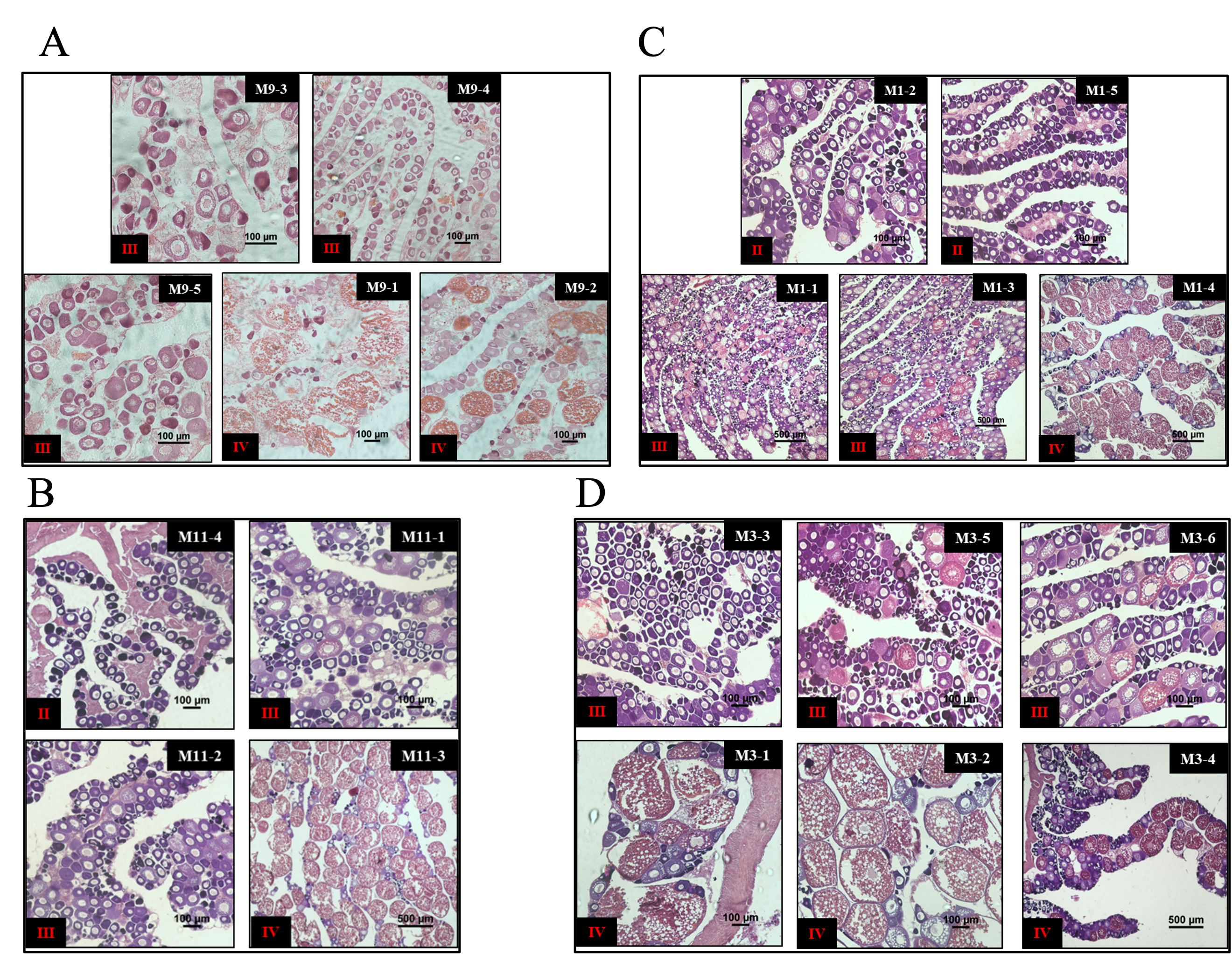

Supplement: Supplementary file 1 [file animals-16-00748-s001.zip › Figure S2.png]

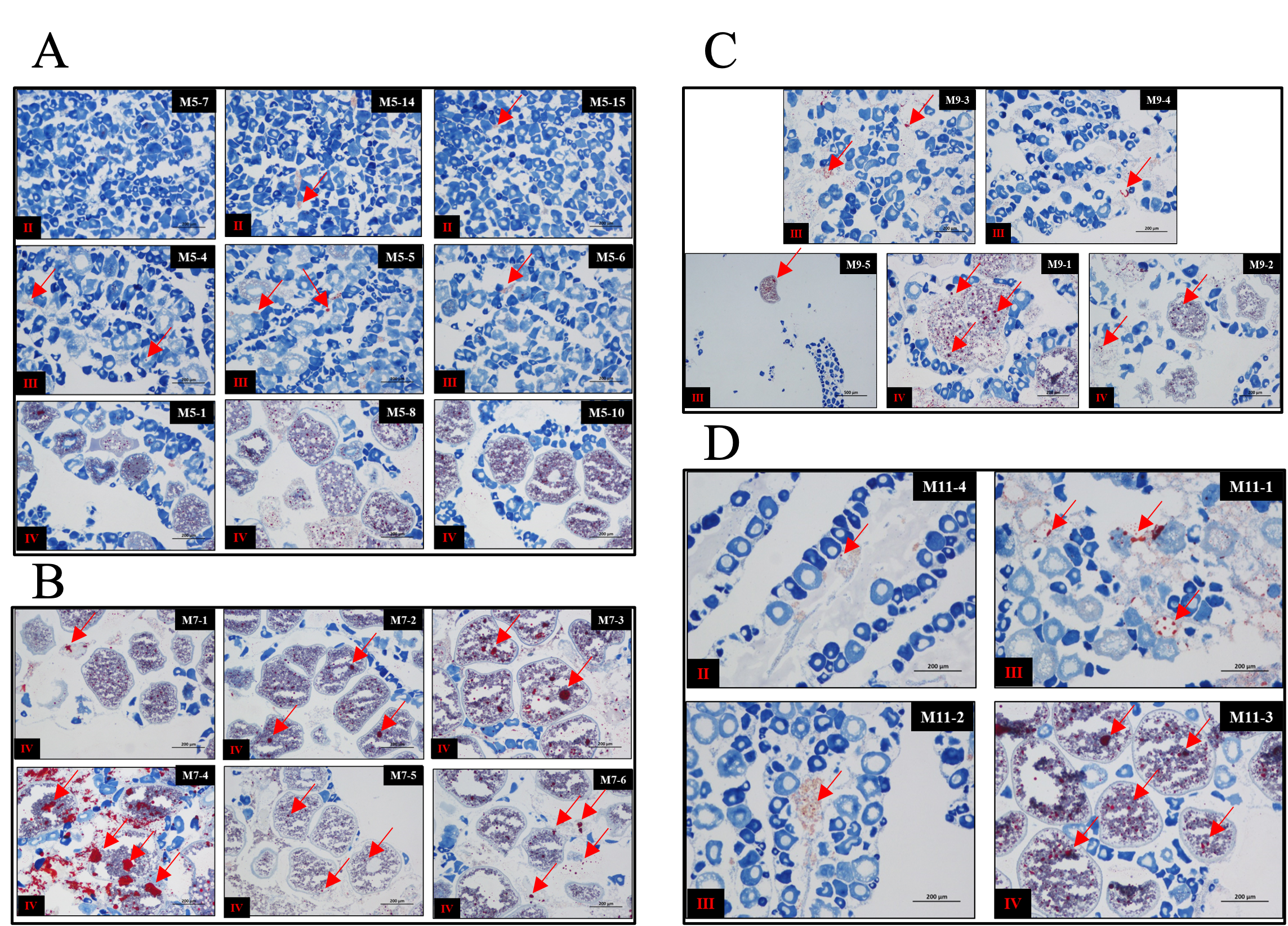

Supplement: Supplementary file 1 [file animals-16-00748-s001.zip › Figure S3.png]

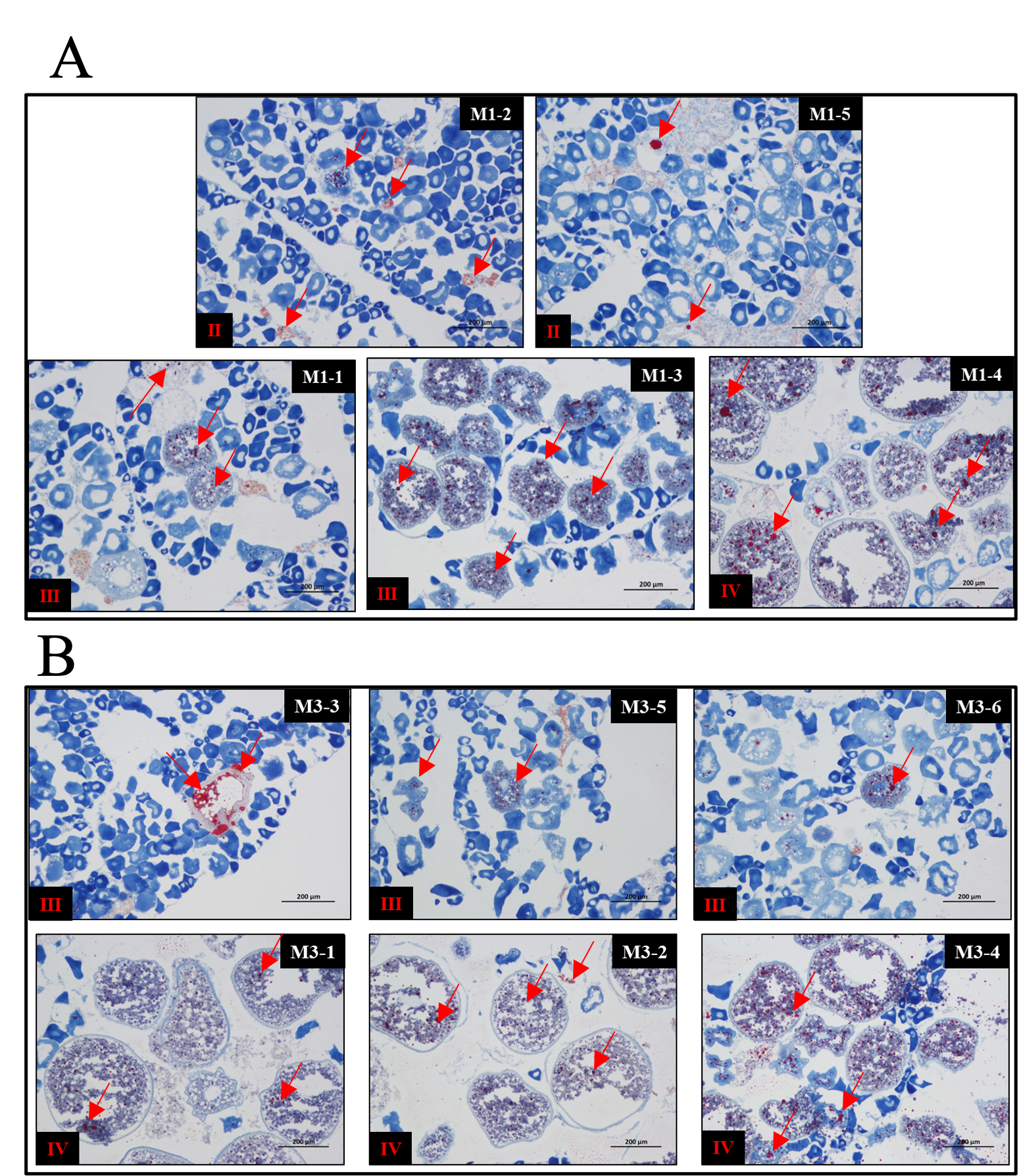

Supplement: Supplementary file 1 [file animals-16-00748-s001.zip › Figure S4.png]

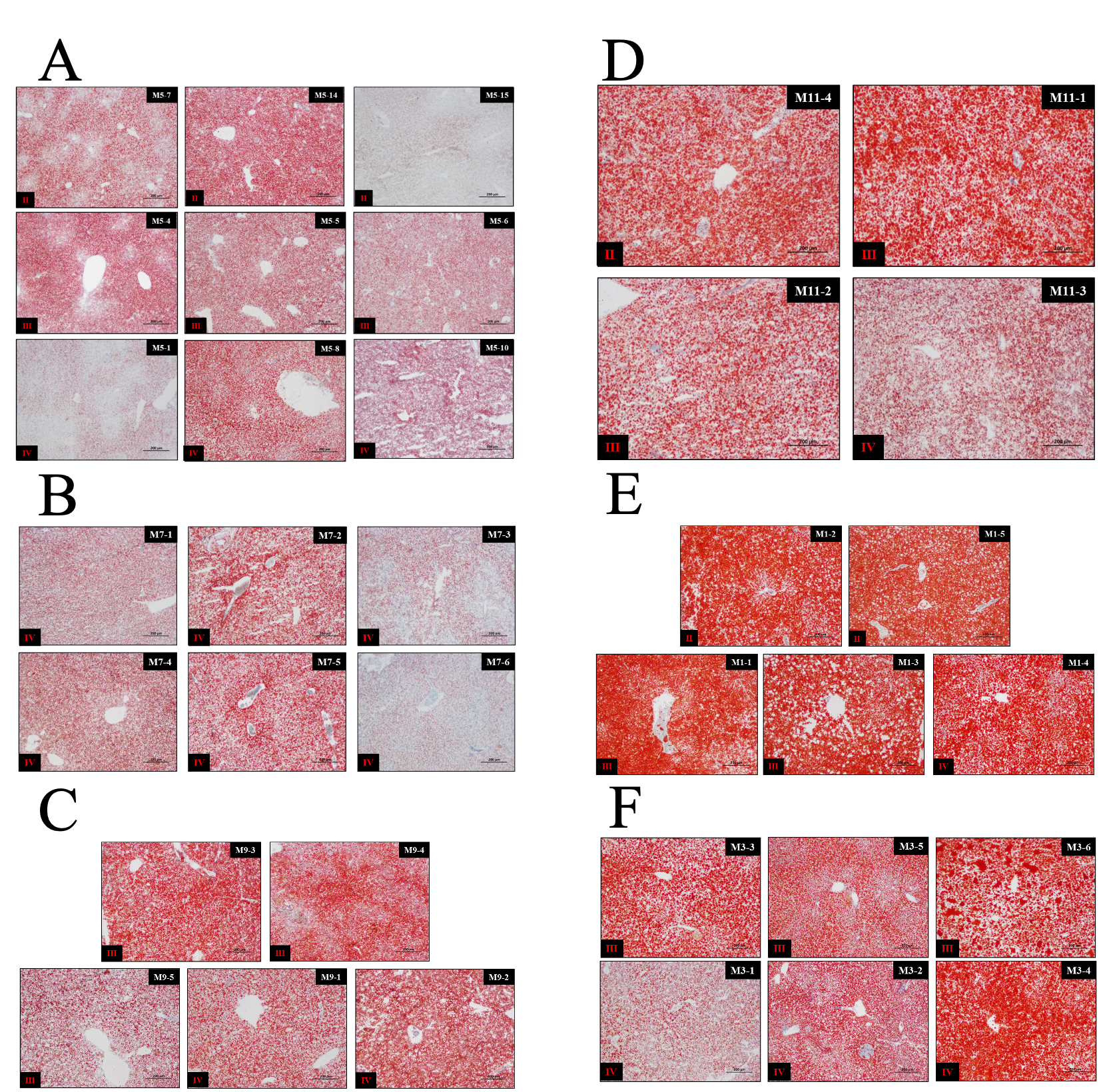

Supplement: Supplementary file 1 [file animals-16-00748-s001.zip › Figure S5.png]
